# Random Forest Predicts Human Ratings of Creative Stories Using Very Small Training Samples

**DOI:** 10.3390/bs16040576

**Published:** 2026-04-11

**Authors:** Baptiste Barbot, Thomas Calogero Kiekens

**Affiliations:** 1 Psychological Sciences Research Institute, Université Catholique de Louvain, 1200 Brussels, Belgium; thomas.kiekens@student.uclouvain.be; 2 Child Study Center, Yale School of Medicine, New Haven, CT 06510, USA

**Keywords:** machine learning, creativity assessment, consensual assessment technique, Random Forest

## Abstract

The Consensual Assessment Technique (CAT) is a gold standard of creativity assessment which provides valid product-based creativity scores that are contextually grounded (stemming from raters with unique expertise, culturally and historically situated). However, its implementation is often demanding (raters’ burden, complex rating designs). This study investigates whether machine learning can effectively simulate expert-panel judgments of creativity using minimal training data. Using a dataset of 411 short stories, we compared the performance of Random Forest (RF), Gradient Boosted Trees, and Decision Tree models, based on story length and Divergent Semantic Integration, to predict expert CAT ratings by (1) identifying the optimal algorithm and (2) the minimum training sample size required for reliable prediction. Results indicate that RF consistently outperformed other algorithms, achieving high correlations with CAT scores (*r* = 0.80) using as few as 25 training stories. Furthermore, RF demonstrated superior accuracy and lower reliance on story length compared to LLM-based scoring models. These findings provide a robust proof-of-concept for using simulated expert panels as a scalable alternative to (decontextualized) automated assessment methods, while reducing human raters’ burden and the logistical constraints of complex rating designs. Extension of this work to different contexts, creativity tasks and domains are necessary to gauge its generalizability.

## 1. Introduction

Creativity is the ability to produce work that is both original and adapted to the domain and/or task constraints (Runco & Jaeger, 2012). With its increased recognition as a 21st century skill and its integration in large-scale assessment such as the Programme for International Student Assessment (PISA; Barbot & Kaufman, 2025; OECD, 2024), creativity becomes a high priority resource to develop and stimulate. However, measuring creativity is challenging and scoring creativity is notoriously cumbersome (Barbot et al., 2019). In recent years, creativity studies in general and creativity assessment in particular have benefited from increased cross-pollination of computer sciences and advances in machine learning (ML).

### 1.1. Creativity Assessment with the Consensual Assessment Technique (CAT)

Historically, and still in current practices, a classic way to assess creative potential is through tasks that simulate the creative work (Barbot et al., 2015) in a broad range of situations such as creating an original recipe (Pichot & Bonetto, 2023), a musical composition (Barbot & Lubart, 2012), a short story (e.g., Johnson et al., 2023; Taylor et al., 2021), or a three-dimensional graphic composition in virtual reality environments (Barbot et al., 2023). The Consensual Assessment Technique (CAT; Amabile, 1982) is a “gold standard” for measuring the creativity of productions completed in such assessments (Cseh & Jeffries, 2019). A caveat to this approach is that creative products are indicative of their author’s creative potential (Hennessey, 1994). Indeed, these situations involve many individual and environmental resources (cognitive abilities, personality traits, environmental enablers) involved in the production of an original and adapted output in the task at hand.

Consistent with social and socio-cultural theories of creativity (Amabile, 1983; Glaveanu et al., 2020), a fundamental assumption of the CAT is that gauging the creative value of a production requires a social consensus. What was once considered creative is perhaps now mundane, and what is considered bizarre today (i.e., highly original but not appropriate) may be eminently creative in the future. In short, there are no absolute norms to determine the creativity of a given output, and the best approximation of its creativity may be captured through subject-matter (human) expert ratings, that are therefore situated in a given historical and temporal context (e.g., Rohrmeier, 2022). Operationally, the CAT consists of rating the creative quality of outputs completed in standardized conditions, by at least three subject-matter experts (Kaufman et al., 2009). Raters typically assess independently the whole set of creative outputs collected in a study, presented in random order using a Likert-type scale ranging from 1 “not at all creative” to 7 “highly creative” (Amabile, 1982; Cseh & Jeffries, 2019; Hennessey, 1994). Statistical consensus is measured by inter-rater reliability coefficients, and if an acceptable consensus is reached (i.e., reliability > 0.70), these subjective ratings can be aggregated into a composite “creativity” score for use in creativity research as well as psychological and educational practice (Barbot et al., 2015).

### 1.2. Limitations and Extensions of the CAT

Beyond methodological challenges surrounding the CAT including the influence of raters’ personality features as well as the type, and years of expertise (Cseh & Jeffries, 2019; Kaufman et al., 2009; Tan et al., 2015), one of its major limitations is that it is quite burdensome for human raters. Finding experts to rate hundreds of artworks, or thousands of first-grade college essays, has its challenges. Indeed, rating creative productions is demanding and can easily lead to raters’ burden (Buczak et al., 2023; Taylor et al., 2021). This is particularly salient with creative writing productions. Perhaps due to these difficulties, creativity assessment has long intimidated many researchers, practitioners and stakeholders, and has generally been neglected as part of a broader psychological and educational assessment (Barbot, 2019).

Recently, two main strategies (not necessarily mutually exclusive) have emerged in creativity studies to overcome these practical limitations, opening new avenues for a broader integration of creativity in assessment practices. The first consists of reducing raters’ burden by optimizing the rating design (i.e., planned missingness design, e.g., Barbot, 2020; Forthmann et al., 2025; Fürst, 2020; Graham, 2009; Graham et al., 2012). Accordingly, raters are each presented with a subset of the sample of production to be rated (with sufficient overlap), such that it is possible to statistically recover the “would-be” values of unrated productions with high accuracy (Forthmann et al., 2025). Approaches for data recovery are numerous, ranging from Full Information Maximum Likelihood (FIML, Mazza et al., 2015)—which does not actually recover missing values but makes use of the full information available to make inferences on parameters of interests—Multiple Imputations (MI, e.g., Forthmann et al., 2025), or machine learning (ML) approaches such as Random Forest (RF, e.g., Buczak et al., 2023) which can work with small datasets, even when the number of predictors exceeds the number of observations (e.g., Biau & Scornet, 2016). Though the planned missingness design approach is effective, the burden somewhat carries over to the researcher that must meticulously organize the rating work to ensure sufficient ratings overlap and accurate recovery of missing values, regardless of the recovery method employed (Forthmann et al., 2025). It also implies that the researcher is working with a finalized dataset: any creative production collected later or obtained from separate studies would require establishing a new rating plan.

### 1.3. Automated Creativity Assessment

The second broad strategy to reduce the limitations of the CAT, which decomposes into several approaches, is automated scoring approaches (Johnson et al., 2023; Luchini et al., 2025; Organisciak et al., 2023; Patterson et al., 2023a, 2023b). This strategy is appealing as it can virtually eliminate the necessity to rely on subject-matter experts to obtain creativity scores which are nevertheless highly predictive of human judgments. Efforts along this line include the Divergent Semantic Integration approach (DSI, Johnson et al., 2023), a proxy for creativity measuring the average semantic dissimilarity in text corpus, or more recently, methods based on Large Language Models (LLMs) such as the Multilingual Assessment of Short Stories (MAoSS; Luchini et al., 2025). However, several limitations surround this approach. The first is that current efforts in automated scoring mainly address written productions, though automated assessments of graphic outputs are increasingly surfacing and demonstrate convincing performance (Acar et al., 2025; Barbot, 2018; Cropley & Marrone, 2025; Patterson et al., 2023a). A second limitation is more conceptual in nature. When automated assessment is based on semantic distance or similar meta-features of text content, the construct measured is distinct from what humans rate with the CAT. More importantly, automated scoring essentially sets a scoring model which becomes the “absolute norm” for determining the creativity of a production. This stands in striking contrast with the fundamental assumption of the CAT: the need for a social consensus to gauge creativity, which is necessarily relative to a given historico-temporal context (Amabile, 1982; Glaveanu et al., 2020; Rohrmeier, 2022). Further, as experts with different kinds of expertise provide different judgments on creativity (e.g., Barbot et al., 2012; Tan et al., 2015), a “generic” algorithm or norm for creativity will not capture the nuances of domain-specific experts.

### 1.4. Present Study

The present study aims to evaluate an alternative approach to address the shortcomings of the CAT procedure, which combines the advantages of planned missingness designs (i.e., reduced raters’ burden) and automated assessments, without overreliance on the “absolute norm” set by the latter. Rather, it aims to develop a panel-specific prediction model (i.e., “simulated expert panel”), such that the would-be values given by that panel to a creative production may be recovered in any data missingness scenario. Specifically, it seeks to use the least possible training material (i.e., only a few human judgments are needed) as well as meta-features extracted from creative productions (e.g., DSI, story length in creative writing) to model the expert panel with high accuracy. The practical advantages of doing so are multiple: (1) it prevents raters’ burden by only involving them in performing the CAT on very small training samples; (2) it allows to apply the simulated expert panel model for scoring productions obtained at later occasions (e.g., in longitudinal studies) rather than soliciting the same panel, or to score different samples (e.g., from different studies) using the same simulated expert panel—a case scenario in which planned missingness cannot be implemented post hoc (Golab & Barbot, 2024); and (3) it does not solely rely on (non-human) “generic” creativity criteria (e.g., DSI, LLMs). In sum, this approach is sought to address many real-life methodological challenges and data scenario issues in creativity studies involving production-based assessment.

In sum, the present study addresses the general research question: can machine learning effectively simulate human judgments of creativity using limited training samples? This objective is decomposed into two main aims. First, we tested the effectiveness of different ML algorithms (namely Random Forest (RF); Gradient Boosted Trees (GBT); and Decision Tree (DT) to predict human ratings of creative stories in different conditions (i.e., size of training production sample, different task prompts, level of prediction [individual raters vs. panel] and data scale [ordinal or continuous]). Second, using the best configuration identified (i.e., algorithm choice, level of prediction and scale), we aimed to evaluate the optimal size of training samples to obtain a viable simulated expert panel. Together, this study sought to provide a proof-of-concept of the feasibility and performance of such “simulated expert-panel” approaches for applications in creativity studies and assessment practices.

## 2. Materials and Methods

### 2.1. Data Source

The source data used in this study consists of a sample of written creative productions using the storyboard task (Golab & Barbot, 2024; Taylor et al., 2021; Taylor & Barbot, 2024). In this task, participants are prompted to create a short story using three pictures that must be used to illustrate the beginning, middle, and end of the story, respectively. Four items (i.e., different sets of picture stimuli) were used and administered to samples of young adults with diverse backgrounds (Golab & Barbot, 2024; Taylor et al., 2021), yielding a total of 411 stories distributed across the four items (see Table 1). This corpus was entirely rated using the CAT by three experienced raters with a high level of inter-rater agreement (Golab & Barbot, 2024). All stories and source data used in this article are available on OSF (https://osf.io/xcrz5 (accessed on 4 April 2026)).

### 2.2. Meta-Features Extraction

Consistent with the DSI approach (Johnson et al., 2023), DSI metrics were extracted for all 411 stories using five distinct linguistic corpuses and embeddings (including TASA and Glove), as well as their average (i.e., general index of semantic integration), using the DSI webapp (Johnson et al., 2023). Additionally, given the strong relationship between story length and human-rated creativity (e.g., Taylor et al., 2021), the word count for each story was also extracted.

### 2.3. Procedure and Data Analytic Plan

We address the first aim (i.e., identification of the best configuration to derive the simulated expert panel) by training models systematically and comparing their performance against human judgments (CAT scores). Specifically, each model was built using CAT individual and aggregated (consensual) ratings, as well as all meta-feature variables. The various configurations were determined as follows. First, for each storyboard item, two missingness scenarios were configured: one with 25% missing values (75% of the sample used for training), and one with 50% missing values (50% used for training), as reflected in Table 1. Second, three ML algorithms were trained for each missingness scenario and storyboard item: Random Forest (RF), Gradient Boosted Trees (GBT), and Decision Tree (DT). Third, models were either trained to predict rater-level response, or panel-level scores. In the case of the former, one model was therefore implemented for each rater, and the resulting predicted values across raters were then aggregated to reconstruct the panel-level score. Fourth, when predicting at the rater level, algorithms were either treating input ratings as ordinal, or as discrete.

In turn, 168 models were developed to address each condition, and the resulting predicted values (i.e., values obtained for those productions that were not included in the training sets) were correlated to the corresponding observed values obtained from the original CAT ratings.

After determining the best configuration (best algorithm to predict CAT scores based on either rater-level—ordinal or discrete—or panel-level set-up), we conducted a second stage of analyses to further evaluate that configuration. To do so, we used the entire production corpus (i.e., *n* = 411 across all 4 storyboard items). From this corpus, we built a validation sample of 32 stories (i.e., productions for which values were to be predicted) selected quasi-randomly. Specifically, within each story set (i.e., 4 storyboard items), 8 stories were randomly chosen with the constraint of selecting four positively rated (i.e., above the mean rating) and four negatively rated (i.e., below the mean rating) stories. This resulted in an approximately normal distribution of the CAT scores for the 32 stories included in this target set (Shapiro–Wilk W = 0.944, *p* = 0.10, skewness = 0.55, kurtosis = 0.74; see ESM, Supplementary Materials). The remaining 379 stories were used to randomly draw different training sets ranging from 10 to 100 stories with increments of 15 stories, then ranging from 100 to 300 stories with increments of 25 stories. In all, these 15 different training scenarios were replicated 10 times (i.e., 150 models were tested under this study aim), each replication using another random set of stories as training material.

As in the first analytical set, all predicted values were correlated to the actual CAT scores to evaluate the models’ ability to capture between-individual differences. Additionally, Mean Absolute Error (MAE) and Root Mean Squared Error (RMSE) were computed to assess the models’ accuracy in terms of predicting absolute score values. For benchmarking purposes, correlations were also computed between the target CAT values (i.e., validation sample) and three comparison benchmarks: story length alone (quartiles of word count to address non-normality and outliers), the DSI alone (average metric), and the scores obtained with the LLM-based Multilingual Assessment of Short Stories (MAoSS; Luchini et al., 2025).

Across all analytical sets, a threshold of *r* = 0.70 (between predicted values and CAT scores) was used as a target for acceptable performance, consistent with common practice in inter-rater reliability, where this value represents a practical lower bound for making group-level inferences (e.g., Kline, 2000). All models were run using Yggdrasil Decision Forest (YDF) as implemented in Simple ML for Sheets (Guillame-Bert et al., 2023). The panel-level models used nine predictors (i.e., five DSI variables, word count, three raters’ variables) and one dependent variable (composite CAT scores). The rater-level models used eight predictors (DSI, word count and the two raters’ variables) and used the target rater variable as the dependent. In this scenario, three models (one per rater) were therefore developed, and the predicted values for each model (or rater) were then aggregated for comparison with the observed composite CAT scores.

## 3. Results

### 3.1. Identification of the Best Prediction Set-Up

Table 2 presents the main findings of the first analytical set in an aggregated fashion. Specifically, it summarizes the average correlations obtained between predicted values and observed CAT scores for both missingness scenarios (25% vs. 50%), aggregating results for all four storyboard items, as a function of the algorithm (DT, RF, GBT), and the level of analysis (panel-level or rater-level, whether using ratings as ordinal or discrete). As shown, the overall pattern of finding suggests that both the GBT and RF algorithms perform rather well in predicting the CAT scores (*r*s ranging 0.66–0.76 across scenarios), largely outperforming DT (mostly <0.50).

A closer look at the algorithm comparison shows that, with RF, panel-level predictions are generally better than (aggregated) rater-level predictions, whether using ratings as ordinal or discrete input data. The opposite pattern is observed with GBT. This is an important finding in favor of RF, outlining the good performance of the simplest set-up (i.e., panel-level prediction), which is less cumbersome to implement than the rater-level set-up. The latter involves the development of one prediction model per rater, and the further aggregation of predicted values for each simulated rater. 

Expectedly, the scenario with less missing data (i.e., more training material) yields better predictions, though the difference in average prediction quality appears small. Overall, RF as predictor of panel-level creativity appears sensibly superior to GBT, with average correlations with CAT scores reaching 0.76, a very satisfactory level of overlap with human ratings (i.e., superior to the typical intercorrelation between raters; Kaufman et al., 2009). Based on this, the identification of the superiority of RF and the parsimony of the panel-level implementation, we selected RF for further evaluation.

### 3.2. Minimal Training Sample Size Needed with RF

**Figure 1:**
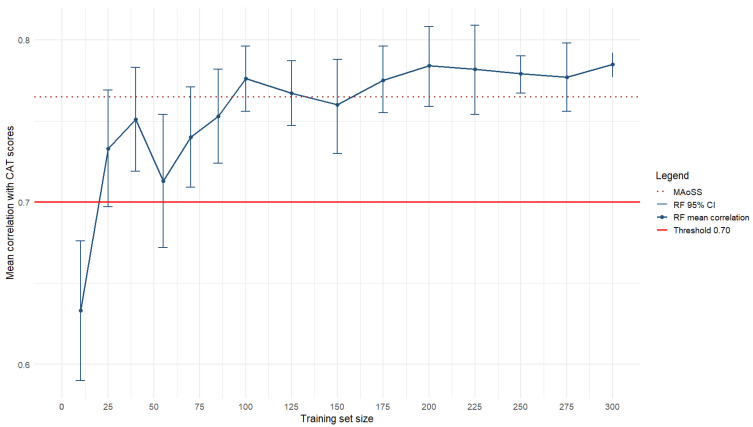
Mean correlations between RF predictions and CAT scores across 15 training scenarios with increasing training sample sizes. Note: MAoSS is a constant as it does not use training data and is considered a benchmark. Y axis truncated for readability.

Table 3 presents the minimum, maximum, standard deviation and mean (and 95% confidence interval) correlations calculated across 10 replications (i.e., random training samples) for the 15 training sample sizes investigated, along with the reliability estimate of the 10 replications. First, our findings suggest very stable predictions (Cronbach *α* > 0.92 over 10 replications), with increasing accuracy (increased *α* and decreased SDs) as the training sample size increases. Expectedly, the overall trend shows an increase in the quality of prediction (i.e., mean CAT correlation with the RF models) with an increased sample size in the training set. Starting with a respectable average prediction of 0.63 with only 10 observations used for training the model (range 0.52 to 0.69), the models stabilize rapidly to the 0.75–0.78 range on average, with training sets ranging from 40 stories to 300 stories. As also illustrated in Figure 1, the gain between 40 and 300 training stories is quite marginal, with an increase of only 3.4% in explained variance in the CAT scores across conditions. In other words, very little improvement in the model prediction quality is observed above 40 training stories. Noticeably, the replication values suggest that the maximal predictions obtained reach 0.80 using only 25 stories. These values stay rather stable (ranging 0.79–0.85) across training sets (i.e., 25 to 300). With this in mind, Figure 1 also shows that, while the average value for prediction exceeds the threshold of *r* = 0.70 with only 25 training stories, the minimum prediction observed across the 10 replications only reaches this threshold stably after 85 training stories.

Table 3 also presents model accuracy indices for predicting absolute point-estimates (MAE and RMSE) computed on z-scored variables across all training set conditions. As shown, both MAE and RMSE indicate only moderate absolute accuracy for the RF-simulated expert-panel approach. While performance improves noticeably between models trained on 10 versus 25 stories, only limited gains are observed in subsequent simulations, with virtually no additional improvement once models are trained on 100 stories or more. Across simulations, MAE values remained in the 0.50–0.55 range, and RMSE values in the 0.65–0.75 range, indicating that absolute point-estimate prediction accuracy plateaus at a moderate level (i.e., on average, predicted values deviate from observed scores by roughly half to three-quarters of a standard deviation).

### 3.3. Comparison with Benchmark Metrics

Table 3 and Figure 1 present the performance accuracy values (correlation with CAT, as well as MAE and RMSE values) for the three benchmark metrics, that is, story length alone, DSI alone, and MAoSS. Correlations with human judgments (CAT) calculated with the whole sample (*n* = 411) were *r* = 0.703, 0.169 and 0.765, respectively. For context, the same correlations calculated with the validation sample alone (*n* = 32) were *r* = 0.693, 0.128 and 0.795, respectively. Given the low predictive power of DSI alone, it was further disregarded as a benchmark to gauge the performance of the RF models. However, given the strong correlations observed between CAT scores and story length (i.e., *r* = 0.703), we further examined the potential confounding effect of length in the relationship between the RF models’ (and MAoSS’) predicted values and CAT scores. Table 3 presents the partial correlation coefficients calculated between the predicted values obtained with the various RF simulation sets and CAT scores (and their bootstrapped 95% confidence intervals), controlling for story length. This approach was chosen (over using residualized CAT scores for story length) because it removes the variance associated with story length from both variables simultaneously (CAT and the RF predictions), thereby directly addressing the substantive question of whether RF predictions relate to the CAT independently of length^1^. With *r* values ranging from 0.527 to 0.743 (mean 0.598), this analytical set suggested that the RF-based simulated expert-panel approach models expert judgments of creativity beyond the mere story length classically embedded in human ratings (Fleckenstein et al., 2020; Liimatta, 2024; Taylor et al., 2021). Interestingly, this analytical set showed that, once story length was controlled for, the RF models remained consistently more strongly correlated with CAT scores than MAoSS was (*r* = 0.446).

In terms of predicting between-individual differences (i.e., correlations with CAT scores), MAoSS performs remarkably well and exceeds the average RF-CAT correlations in simulations using up to 100 training samples, although the magnitude of these differences is small and not statistically significant. In contrast, for absolute point-estimate accuracy, the MAE and RMSE values obtained for MAoSS indicate that the RF approach achieves better absolute prediction performance, with this advantage becoming consistently apparent once 85 training samples are used.

## 4. Discussion

The present study introduced and tested an alternative approach to creativity assessment designed to overcome several practical limitations of the traditional Consensual Assessment Technique (CAT). By combining advantages and principles of planned missingness designs and machine learning (ML)-based prediction, we sought to reduce raters’ burden while preserving the interpretive specificity of (human) expert-panel judgments. Using the storyboard tasks as a testbed, the findings provide a solid proof-of-concept for developing “simulated expert panels” models capable of reproducing human rating patterns under various data conditions, at least as it applies to creative writing and the storyboard task specifically. In all, over 300 models were developed to assess the potential of this approach.

### 4.1. Predictive Performance of Different ML Algorithms

Under our first research aim (Aim 1), 168 models were compared to gauge the predictive performance of different ML algorithms (RF, GBT, DT) under varying conditions (training sample size, prompt, rating scale, level of prediction). Results indicated that both Gradient Boosted Trees (GBT) and Random Forest (RF) algorithms achieved strong predictive accuracy, with correlations between predicted values and observed CAT scores ranging from *r* = 0.66 to 0.76 across missingness scenarios. In contrast, Decision Tree (DT) models performed notably worse (typically *r* < 0.50), suggesting a limited suitability for this task. Closer examination revealed that, for RF, panel-level predictions consistently outperformed (aggregated) rater-level predictions, regardless of whether ordinal or discrete rating scales were used. The opposite pattern was observed for GBT, where rater-level models yielded slightly better results. This distinction favors the RF algorithm, as its simpler panel-level implementation offers comparable or superior performance with substantially reduced modeling complexity—requiring only one prediction model (i.e., panel-level) rather than one model per rater. As expected, the scenario with less missing data (25%) produced better prediction accuracy than the 50% missingness condition, although differences were modest. Overall, the RF models predicting panel-level scores achieved the highest correlations with CAT ratings (up to *r* = 0.76), exceeding typical inter-rater reliability benchmarks. Accordingly, RF with panel-level prediction was selected for further examination.

The consistently high predictive accuracy of the RF algorithm, coupled with its stability across data conditions, suggests that expert consensus patterns can be effectively captured and generalized using relatively small samples of human-rated creative writing productions and limited meta-feature extraction (at least with the storyboard task; Taylor et al., 2021). Importantly, the superior performance of the simpler panel-level RF models—compared to more complex rater-level configurations or Gradient Boosted Trees (GBTs)—underscores the practicality and efficiency of this approach. This indicates that interpretable expert-like evaluations can be achieved without replicating individual raters’ idiosyncrasies, while still preserving the collective “voice” of the panel, consistent with the basic principles of the Consensual Assessment Technique (Amabile, 1983; Hennessey, 1994). However, the relative superiority of RF performance in our study (compared with GBT and DT) may be partly attributable to the limited size of the training sets. Indeed, RF relies on bootstrap aggregation, which reduces model variance through averaging and tends to yield more stable predictions when data is sparse. In contrast, GBTs fit trees sequentially to the residuals, a process that can overfit noise when training samples are limited (unless substantial regularization is applied). The “bagging” approach of RF (i.e., bootstrap aggregation, where multiple models are trained on bootstrapped samples and averaged to reduce variance) reduces the high variability of individual tree models overall (Buczak et al., 2023) by growing trees independently on random subsets of the data, a safeguard that becomes particularly important when working with small datasets. In sum, this first analytical set does not point to the absolute superiority of RF over other ML approaches but rather underscores its suitability for applications similar to those examined here (i.e., training on small samples).

### 4.2. Minimal Training Sample Size Needed for Acceptable Predictive Accuracy

Under our second research aim (Aim 2: identifying the minimal training sample size needed), 150 RF models (i.e., 10 replications of 15 training scenarios with increasing training sample sizes) were developed to determine the minimal viable training sample size to yield acceptable predictive accuracy in terms of between-individual differences (*r* ≥ 0.70). Results showed RF models yielded highly stable predictions across replications (Cronbach’s α > 0.92) and increasing accuracy with larger training samples (i.e., correlation between predicted values and observed CAT values). Prediction quality rose sharply up to about 40 training stories (*r* ≈ 0.75–0.78) and then plateaued, indicating minimal improvements beyond this point. On average, the benchmark threshold of *r* = 0.70 was reached with as few as 25 training stories and achieved consistently across replications with approximately 85 stories.

In terms of absolute point-estimate predictions gauged with MSA and RMSE, our findings suggested only limited accuracy of the RF-simulated expert-panel approach, with deviations from actual values in the 0.50–0.70 standard deviations range. These findings suggest that although the simulated expert-panel approach reliably captures relative differences in human creativity rating scores, its ability to generate precise point-estimates remains somewhat limited. In practice, this implies that this approach is better suited for comparative judgments (e.g., research settings with correlational designs) than for applications requiring highly accurate individual-level score reconstruction (e.g., clinical settings).

Comparisons with benchmark variables (story length, DSI, the MAoSS) indicated that, in terms of predicting relative differences between individuals, the RF model’s performance closely matched that of MAoSS, an LLM-based algorithm to score short stories (Luchini et al., 2025), while far exceeding that of DSI (Johnson et al., 2023), relying on semantic distance only. However, further analyses revealed that MAoSS appeared more sensitive to story length than the RF approach was and, to some extent, produced less accurate absolute point-estimate predictions, especially when RF training samples exceeded 85 stories. Overall, these results suggest that a relatively small creative writing training sample using the storyboard task (40–85 stories) is sufficient to achieve reliable and robust expert-panel simulation in terms of predicting individual differences in human creativity ratings, and more accurate point-estimate predictions than “generic” LLM-based scoring methods (i.e., MAoSS), especially when training RF models with 85 stories or more.

### 4.3. Significance and Implications

Together, the present findings provide compelling support for the feasibility of using ML to model expert-panel judgments of creative writing with minimal human input. Methodologically, these results highlight a viable path to reducing the burden (logistical and cognitive) traditionally associated with the CAT. By requiring only a limited number of human-rated samples to train the model, researchers can minimize rater fatigue and resource demands while maintaining satisfactory fidelity to expert judgments (in terms of between-individual differences). The finding that prediction quality stabilizes with as few as 40 to 85 training stories reinforces the scalability of this method for larger studies (e.g., longitudinal studies), where repeated expert ratings are often impractical (Barbot, 2019). Furthermore, the ability to reproduce panel-level judgments across studies or measurement occasions (e.g., longitudinal designs) extends the CAT’s applicability to contexts where planned missingness designs cannot be easily implemented post hoc (Golab & Barbot, 2024). Theoretically, the study demonstrates that computational models can approximate, rather than replace, human expertise—emulating the evaluative structure of a specific expert panel rather than imposing a universal or “generic” norm of creativity assessment. This distinction is critical, as it preserves the contextual, domain-sensitive and “human-grounded” nature of creativity assessment. Beyond its immediate methodological utility, this approach opens new avenues for studying creative development over time and across domains, where consistent, scalable, and contextually grounded measures of creativity are most needed. Given the nature of our study (i.e., proof-of-concept), this conclusion should, however, be taken as applying specifically to short creative writing samples (here, the storyboard task), before it can be generalized to other tasks, domains and contexts.

### 4.4. Limitations and Future Directions

While the present study provides a strong proof-of-concept for modeling expert-panel judgments through RF algorithms in creative writing, several limitations should be noted. First, the validation set used in our second set of analysis (*n* = 32 productions) may have represented a particularly favorable subset for comparison with MAoSS, story length, and/or an unfavorable subset for comparison with DSI. Extending this work by testing the approach to alternative (and larger) validation sets within the same data source would help evaluate the robustness and stability of our findings.

Second, although the study already involved training many models across multiple conditions (over 300 models here), the predictive framework relied on only two main meta-features—story length and indicators of Divergent Semantic Integration (DSI). Expanding the meta-feature space by incorporating a broader range of linguistic, semantic, or structural features—e.g., using computational libraries and frameworks such as the emoatlas (Semeraro et al., 2025) as well as features extracted from other natural language processing or large language models (e.g., Brunato et al., 2020)—could enhance predictive accuracy and help better reproduce the subtleties of creativity captured by human judgements in written productions. Indeed, the creativity of written production goes beyond the mere number of words and their average semantic distance (e.g., Barbot et al., 2012; Golab & Barbot, 2024; Lubart, 2009). Yet, our study shows a very high correlation between story length and human judgment which deserves further investigation. In their validation work, Taylor et al. (2021) suggest that this high correlation may reflect that longer stories give more opportunities for expressing creative ideas, but it might also reflect a possible bias: judges might unconsciously equate more text (or more elaboration) with higher creativity, a consistent bias in human judgments of written productions (Fleckenstein et al., 2020; Liimatta, 2024). This points to another important line of further examination where story length is controlled experimentally, or simulations using random, non-sensical productions of different lengths and average semantic distances are introduced to further gauge the performance of our simulated expert-panel approach.

Third, our RF models and analyses did not explicitly account for stimulus specificity in evaluating performance (i.e., the particular storyboard item used). Although supplementary analyses indicated that including the storyboard item as a predictor did not meaningfully enhance prediction accuracy (see ESM, Supplementary Materials), it remains possible that building item-specific models—rather than treating the item as an additional covariate—could yield better results. Future work should explore this possibility to determine whether the RF-simulated expert-panel approach can be improved in predicting both between-individual differences in human-rated creative writing and the more precise point-estimate values required for high-stakes applications.

Finally, the overall excellent performance of MAoSS in our case scenario raises important questions about the added value (or incremental validity) of the simulated expert-panel approach. While MAoSS (which here leveraged automated assessment of semantic features) achieved correlations with human ratings comparable to those of our RF-based simulated panel, it does so without any (local) human inputs, in a “far-generalization” scenario (i.e., MAoSS is tuned for a specific story-writing prompt quite distinct from the storyboard task), highlighting the efficiency of MAoSS and more broadly the scalability of fully automated approaches. This encourages reflection on the circumstances under which simulating a human panel is truly advantageous: although our approach preserves the contextual and temporal specificity of expert judgments in situations where automated models already approximate human consensus with high fidelity (in terms of between-individual differences), the incremental benefit of incorporating simulated expert-panel predictions may be modest. Keeping the above limitation in mind (our approach was itself “generic” in terms of the item-specificity of the storyboard task), the simulated expert-panel framework retains a conceptual edge by emulating a specific panel’s evaluative standards rather than imposing a generic or universal norm, which may be critical when assessing productions in very specific tasks or domains, across culturally distinct panels—an important source of variation in human judgment (Mourgues et al., 2014; Tan et al., 2015)—or over longitudinal designs where expert judgment consistency is paramount (Barbot, 2019, 2022). Thus, the high performance of MAoSS does not invalidate the utility of our proposed approach but rather situates it within a range of novel approaches, all of which represent a nascent effort to address the historically cumbersome production-based creativity (writing) assessment process.

More broadly, applying the simulated expert-panel approach to different creative writing tasks, creative domains (e.g., visual art, design, music) and larger, more diverse datasets would inform its generalizability. This extension may face current limitations in meta-feature extractions of modalities other than written productions, though progress in characterizing graphic (Patterson et al., 2023a; Zhang et al., 2025) or musical productions (Rohrmeier, 2022) are rapidly developing.

## 5. Conclusions

This study demonstrates the feasibility and utility of simulating expert-panel judgments of creative writing using Random Forest (RF) models. With modest training samples, RF predictions closely approximate human Consensual Assessment Technique (CAT) ratings, reducing raters’ burden while preserving contextual and culturally grounded evaluations, a prerequisite to capture the relativity of creativity (Amabile, 1982, 1983; Glaveanu et al., 2020; Rohrmeier, 2022). Although automated approaches like MAoSS (Luchini et al., 2025) also achieve high correlations with human ratings, they operate as absolute norms and lack sensitivity to temporal, situational, and task-specific context, highlighting a key advantage of human-trained models. Features such as story length and semantic diversity, while predictive, are not sufficient on their own to fully capture creative quality, emphasizing the permanent relevance of human judgment as a reference standard. Overall, this proof-of-concept suggests that combining minimal human input with machine learning offers a practical, scalable, and reliable framework for creativity assessment in many use-case scenarios (e.g., longitudinal investigations), while maintaining alignment with the core principles of the CAT. We encourage readers to extend this work by making use of the publicly available data (https://osf.io/xcrz5 (accessed on 4 April 2026)) and further our approach to simulate expert panels or address new research questions (e.g., excluding length as a predictor, employing alternative algorithms, incorporating new meta-features, or deriving human judgments less dependent on length). Future work extending this RF-based expert-panel simulation approach to other data scenario, creativity tasks and domains are also necessary to gauge the generalizability of this approach beyond the storyboard task used here as a working example.

## Figures and Tables

**Table 1:** Number of creative productions across the storyboard items and simulation scenarios.

		Item 1A (*n* = 102)	Item 1B (*n* = 97)	Item 2A (*n* = 107)	Item 2B (*n* = 103)
25% missingness	Training sample	76	73	80	77
	Predicted values	26	24	27	26
50% missingness	Training sample	51	47	54	52
	Predicted values	51	48	53	51

**Table 2:** Average correlations between predicted values and observed CAT scores across algorithms, levels of analysis, and missingness scenarios.

	25% Missingness Scenarios			50% Missingness Scenarios			All Scenarios		
	GBT	RF	DT	GBT	RF	DT	GBT	RF	DT
Rater-level (discrete)	0.706	0.718	0.510	0.677	0.718	0.439	0.692	0.718	0.479
Rater-level (ordinal)	0.683	0.696	0.483	0.667	0.648	0.414	0.675	0.672	0.449
Panel-level	0.674	0.778	0.517	0.671	0.735	0.375	0.672	0.757	0.470

**Table 3:** Prediction performance of Random Forest models across training sample sizes.

Models	Mean CAT *r*	Min	Max	SD	95% CI Mean CAT *r*		*α*	MAE	RMSE	Partial CAT *r*	95% CI Partial CAT *r*	
					Lower	Upper					Lower	Upper
RF_10	0.633	0.522	0.692	0.060	0.590	0.676	0.920	0.640	0.841	0.604	0.331	0.798
RF_25	0.733	0.635	0.802	0.050	0.697	0.769	0.973	0.551	0.716	0.578	0.252	0.773
RF_40	0.751	0.69	0.829	0.045	0.719	0.783	0.978	0.540	0.692	0.743	0.537	0.879
RF_55	0.713	0.606	0.794	0.057	0.672	0.754	0.977	0.586	0.743	0.597	0.274	0.783
RF_70	0.740	0.641	0.795	0.044	0.709	0.771	0.979	0.555	0.707	0.585	0.240	0.790
RF_85	0.753	0.703	0.813	0.040	0.724	0.782	0.984	0.537	0.690	0.588	0.342	0.749
RF_100	0.776	0.727	0.820	0.029	0.756	0.796	0.982	0.496	0.658	0.675	0.494	0.824
RF_125	0.767	0.720	0.813	0.028	0.747	0.787	0.984	0.521	0.671	0.615	0.285	0.797
RF_150	0.760	0.703	0.828	0.040	0.730	0.788	0.988	0.521	0.680	0.572	0.244	0.751
RF_175	0.775	0.733	0.820	0.029	0.755	0.796	0.988	0.498	0.659	0.623	0.339	0.789
RF_200	0.784	0.723	0.833	0.035	0.759	0.808	0.992	0.497	0.646	0.582	0.244	0.781
RF_225	0.782	0.726	0.849	0.039	0.754	0.809	0.992	0.493	0.648	0.597	0.291	0.781
RF_250	0.779	0.755	0.804	0.016	0.767	0.79	0.994	0.503	0.655	0.555	0.224	0.752
RF_275	0.777	0.719	0.814	0.029	0.756	0.798	0.995	0.504	0.656	0.537	0.207	0.737
RF_300	0.785	0.767	0.804	0.010	0.777	0.792	0.997	0.502	0.652	0.527	0.146	0.760
Length *	0.703	_	_	_	0.652	0.751	_	0.573	0.795	_	_	_
DSI *	0.169	_	_	_	0.084	0.267	_	0.938	1.288	0.138	0.045	0.258
MAoSS *	0.765	_	_	_	0.714	0.808	_	0.541	0.685	0.446	0.364	0.527

Note: RF_10 to RF_300 denotes the number of stories used as training set (i.e., from 10 to 300 stories) across 10 replications. For example, for RF_40, the values reported are those observed with 10 different RF models that have used a random set of 40 training stories. * Estimates obtained on the whole sample (*n* = 411); partial CAT *r* computes the correlation between the target indicator (in rows) and CAT, controlling for story length. MAE = Mean Absolute Error, RMSE = Root Mean Squared Error. 95% confidence intervals (CIs) were calculated using 1000 bootstrap samples.

## Data Availability

The original data presented in the study are openly available on the Open Science Framework (OSF) at https://osf.io/xcrz5 (accessed on 17 October 2024).

## Note

1 For completeness, we examined RF performance using CAT scores residualized for story length. However, this approach has important limitations: residualizing only the CAT removes length-related variance from the criterion variable while leaving it intact in the RF predictions. As a result, the residualized CAT scores represent a transformed outcome (with limited interpretability) that the RF models were not trained to predict. Therefore, this approach does not provide a meaningful evaluation of whether the RF models capture variance in human judgments beyond length. As expected under these conditions, correlations between the RF predictions and residualized CAT scores were low (r ≈ 0.20–0.30). A fuller summary of these exploratory analyses is provided in the ESM.

## Supplementary Materials

The following supporting information can be downloaded at: https://www.mdpi.com/article/10.3390/bs16040576/s1, ESM.

## Abbreviations

The following abbreviations are used in this manuscript:

CAT	Consensual Assessment Technique
DSI	Divergent Semantic Integration
DT	Decision Tree
GBT	Gradient Boosted Tree
FIML	Full Information Maximum Likelihood
LLM	Large Language Model
MAoSS	Multilingual Assessment of Short Stories
MI	Multiple Imputation
ML	Machine Learning
RF	Random Forest
SL	Story length
YDF	Yggdrasil Decision Forest
