# Supplementary material for: Random Forest Predicts Human Ratings of Creative Stories Using Very Small Training Samples
Source: Behav Sci (Basel). 2026 Apr 11;16(4):576. doi: 10.3390/bs16040576 (PMC13114157; doi:10.3390/bs16040576)
Supplement: Supplementary file 1 [file behavsci-16-00576-s001.zip › behavsci-4018428-supplementary.pdf]

ESM

1. Detailed Results of the first analytical set: dentification of the best configuration to derive the simulated expert-panel 1
2. Descriptive statistics for input variables 4
   a. Full dataset 4
   b. Group by set 5
3. Descriptive statistics for Validation sample 7
   a. All selected stories 8
   b. Selected stories grouped by set 9
4. Prediction of CAT using RF 12
   a. R Code to process RF models 12
   b. Figure 1 13
   c. Calculating MAE & RMSE 14
5. Code and outputs for the RF simulations including the Storyboard item as input variables 22
   a. Calculating the correlation with CAT 22
   b. Calculating MAE & RMSE 24
6. Code and outputs for the RF simulations with CAT residuals as output 32
   a. Calculating the correlation with CAT_rez 32
   b. Calculating MAE & RMSE 35

1. Detailed Results of the first analytical set: dentification of the best configuration to derive the simulated expert-panel

		GBT	RF	DT
Item 1A - 25% missingness	Rater 1 - discrete	0.726	0.748	0.57
	Rater 2 - discrete	0.819	0.813	_
	Rater 3 - discrete	0.222	0.286	0.444
	Rater 1 - ordinal	0.758	0.815	-0.277
	Rater 2 - ordinal	0.793	0.749	
	Rater 3 - ordinal	0.315	0.006	0.278
	Composite - discrete	0.842	0.801	0.65
	Composite - ordinal	0.795	0.781	0.253
	Panel - discrete	0.811	0.777	0.572
Item 1A - 50% missingness	Rater 1 - discrete	0.608	0.704	0.408
	Rater 2 - discrete	0.674	0.741	0.35
	Rater 3 - discrete	0.483	0.524	0.563
	Rater 1 - ordinal	0.677	0.623	0.091
	Rater 2 - ordinal	0.716	0.603	0.316
	Rater 3 - ordinal	0.374	0.287	0.499
	Composite - discrete	0.787	0.814	0.756
	Composite - ordinal	0.833	0.705	0.607
	Panel - discrete	0.775	0.806	_
Item 1B - 25% missingness	Rater 1 - discrete	0.497	0.482	-0.033
	Rater 2 - discrete	0.696	0.717	0.509
	Rater 3 - discrete	0.396	0.611	0.519
	Rater 1 - ordinal	0.179	0.941	0.812
	Rater 2 - ordinal	0.983	0.616	0.425
	Rater 3 - ordinal	0.192	0.475	_
	Composite - discrete	0.646	0.698	0.622
	Composite - ordinal	0.746	0.766	0.709
	Panel - discrete	0.67	0.983	0.609
Item 1BA - 50% missingness	Rater 1 - discrete	0.216	0.568	0.051
	Rater 2 - discrete	0.662	0.777	-0.007
	Rater 3 - discrete	0.392	0.61	0.137
	Rater 1 - ordinal	0.326	0.598	0.38
	Rater 2 - ordinal	0.603	0.522	-0.213
	Rater 3 - ordinal	0.419	0.513	_
	Composite - discrete	0.576	0.75	0.044
	Composite - ordinal	0.562	0.661	0.139

	Panel - discrete	0.666	0.829	0.224
Item 2A - 25% missingness	Rater 1 - discrete	0.767	0.711	_
	Rater 2 - discrete	0.629	0.712	0.103
	Rater 3 - discrete	0.283	0.398	-0.066
	Rater 1 - ordinal	0.49	0.487	_
	Rater 2 - ordinal	0.55	0.447	_
	Rater 3 - ordinal	0.164	0.444	_
	Composite - discrete	0.743	0.746	0.153
	Composite - ordinal	0.638	0.694	_
	Panel - discrete	0.733	0.73	0.332
Item 2A - 50% missingness	Rater 1 - discrete	0.466	0.476	_
	Rater 2 - discrete	0.635	0.654	_
	Rater 3 - discrete	0.279	0.18	_
	Rater 1 - ordinal	0.49	0.48	_
	Rater 2 - ordinal	0.49	0.628	_
	Rater 3 - ordinal	0.106	_	_
	Composite - discrete	0.677	0.613	_
	Composite - ordinal	0.648	0.624	_
	Panel - discrete	0.629	0.632	_
Item 2B - 25% missingness	Rater 1 - discrete	0.497	0.658	0.6
	Rater 2 - discrete	0.47	0.543	0.504
	Rater 3 - discrete	0.376	0.444	0.368
	Rater 1 - ordinal	0.455	0.412	0.429
	Rater 2 - ordinal	0.45	0.529	_
	Rater 3 - ordinal	-0.036	0.269	_
	Composite - discrete	0.593	0.628	0.613
	Composite - ordinal	0.551	0.541	0.487
	Panel - discrete	0.48	0.623	0.555
Item 2B - 50% missingness	Rater 1 - discrete	0.471	0.513	0.446
	Rater 2 - discrete	0.65	0.726	_
	Rater 3 - discrete	0.328	0.382	_
	Rater 1 - ordinal	0.53	0.353	0.387
	Rater 2 - ordinal	0.508	0.561	_
	Rater 3 - ordinal	0.119	0.164	0.259
	Composite - discrete	0.669	0.693	0.516
	Composite - ordinal	0.626	0.601	0.496

Panel - discrete	0.614	0.674	0.526

2. Descriptive statistics for input variables

a. Full dataset

Table 1: Descriptive statistics – Full dataset

N	meanCAT	sd	min	max	median
411.00	2.85	1.07	1.00	6.30	2.70

Table 2: Normality tests – Full dataset

Test	Statistic	p_value
Kolmogorov–Smirnov (Lilliefors)	0.109	0.000
Shapiro–Wilk	0.971	0.000

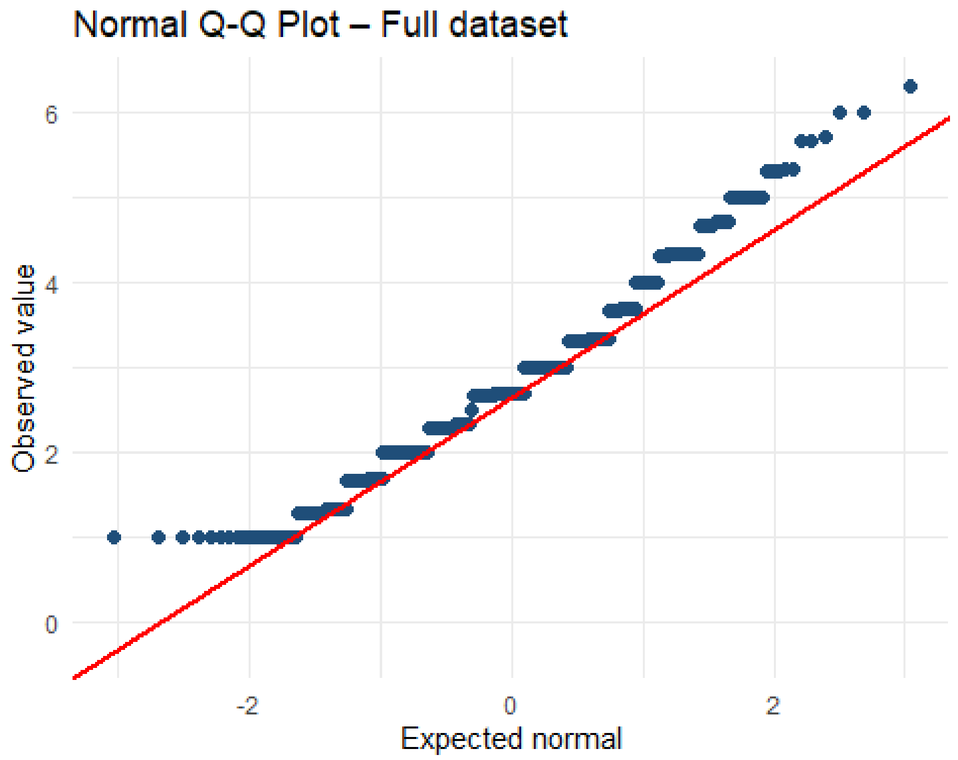

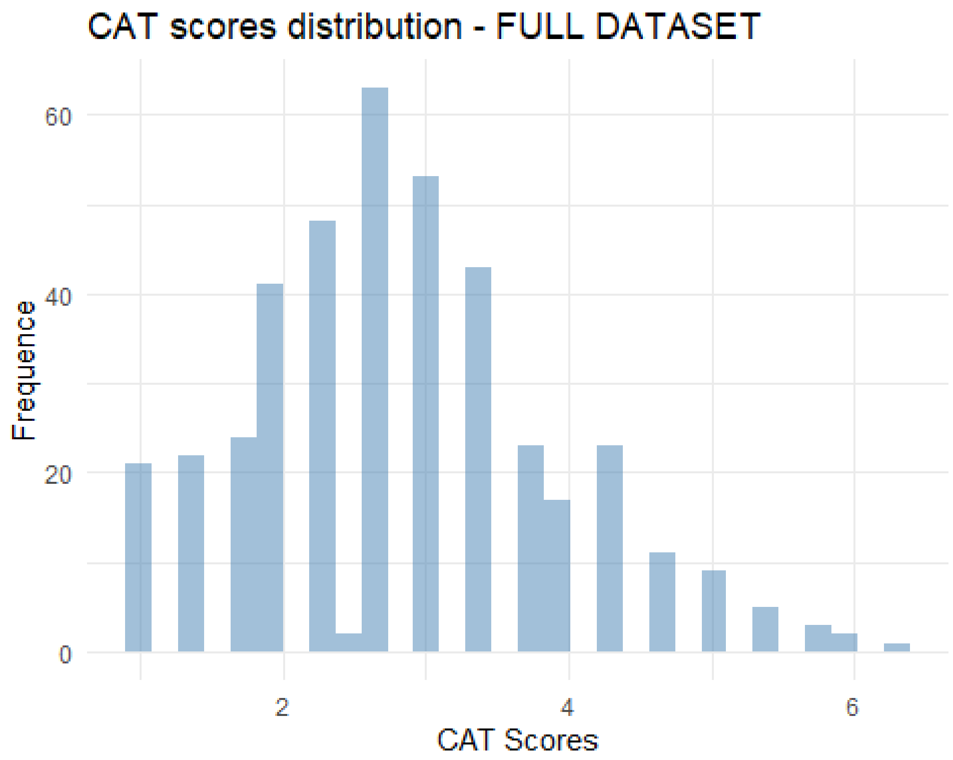

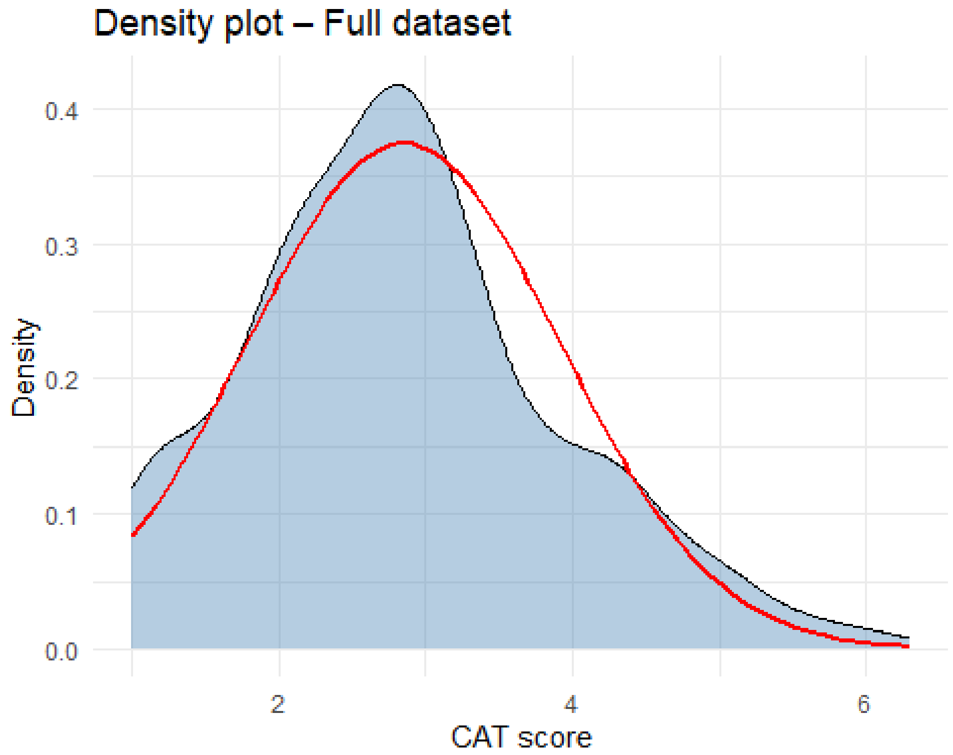

b. Group by set

Table 3: Descriptive statistics – By set

set	N	meanCAT	meanDSI	meanSL	sd	min	max	median
1A	102.00	2.81	0.76	271.21	1.09	1.00	5.33	2.67
1B	97.00	2.89	0.78	280.10	1.23	1.00	5.67	3.00
2A	108.00	2.73	0.76	316.96	0.90	1.00	6.00	2.70
2B	104.00	2.95	0.76	331.12	1.05	1.00	6.30	3.00

Table 4: Normality tests – By set

set	KS	KS_p	SW	SW_p
1A	0.110	0.004	0.962	0.005
1B	0.086	0.075	0.964	0.009
2A	0.136	0.000	0.937	0.000
2B	0.136	0.000	0.958	0.002

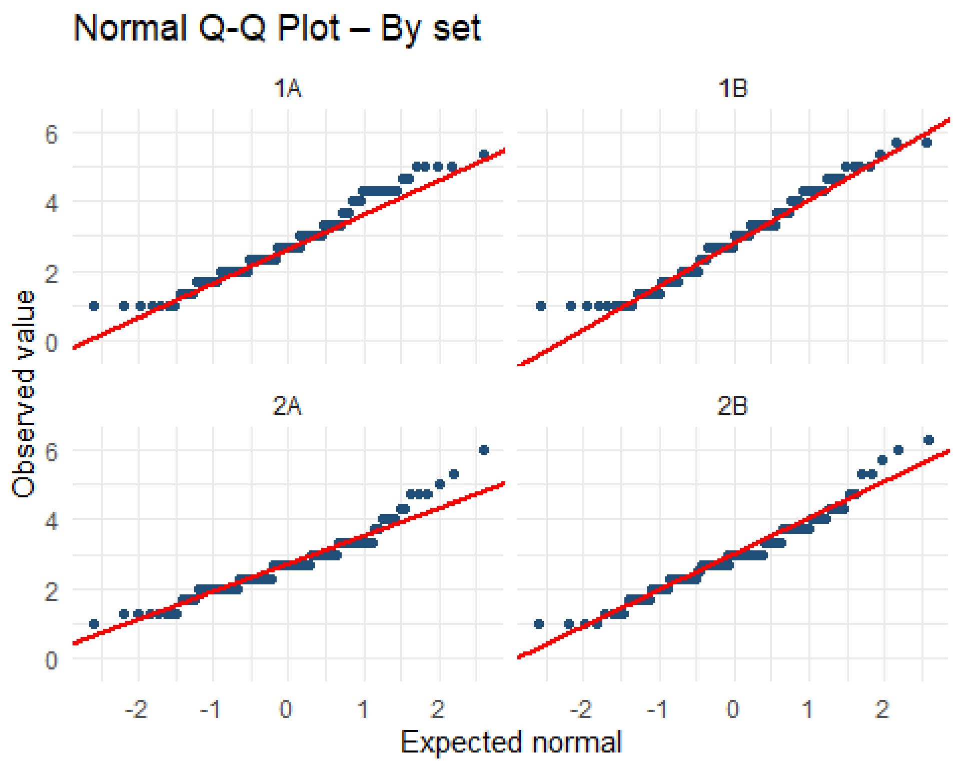

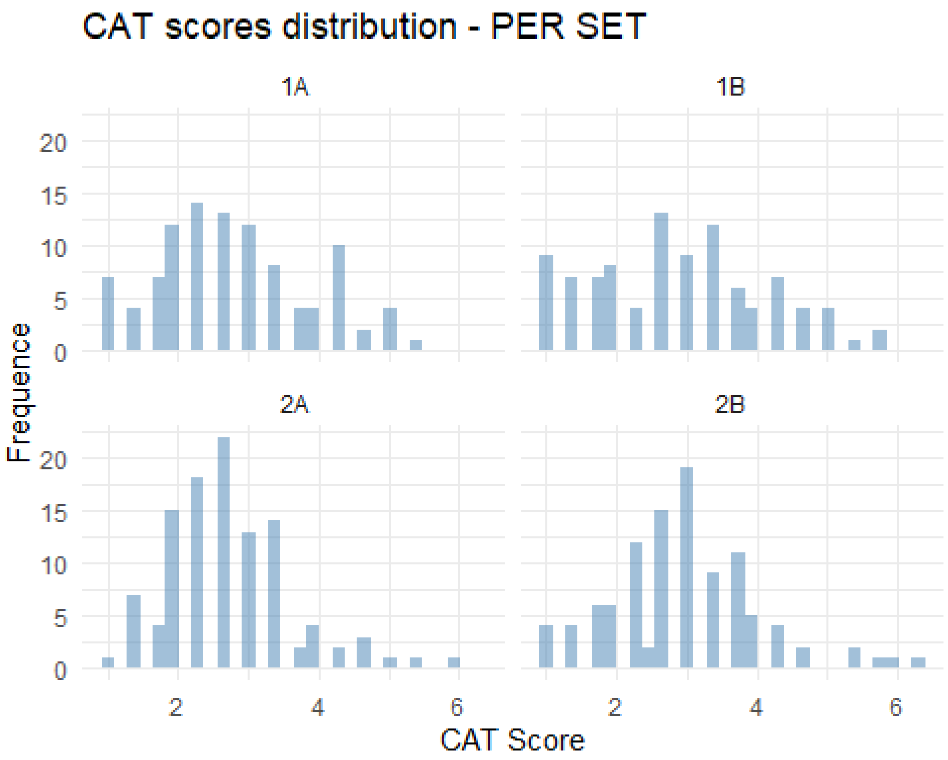

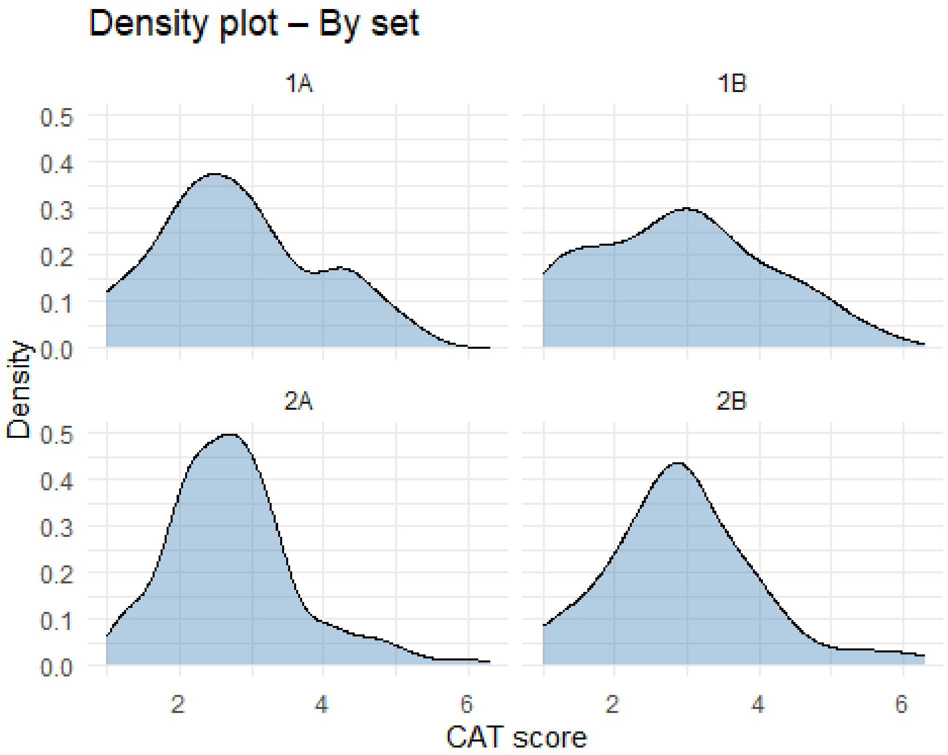

3. Descriptive statistics for Validation sample

a. All selected stories

Table 5: Descriptive statistics – Selected stories

n	meanCAT	sd	min	max	median
32.00	2.91	1.15	1.00	5.70	2.70

Table 6: Normality tests for CAT

Test	Statistic	p_value
Kolmogorov–Smirnov (Lilliefors)	0.138	0.127
Shapiro–Wilk	0.944	0.096

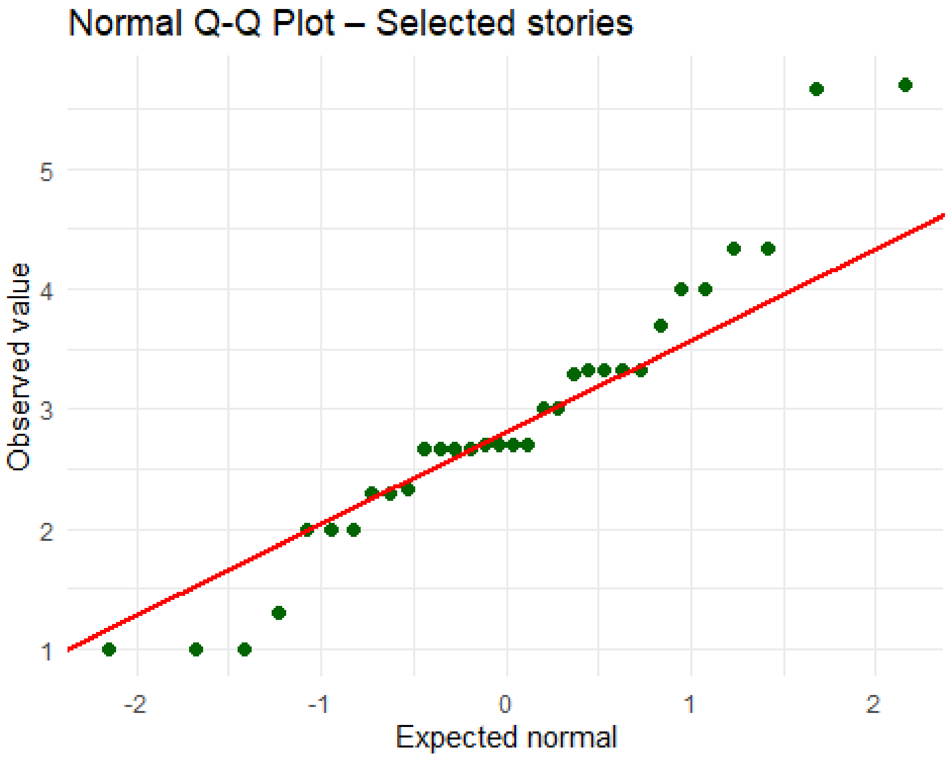

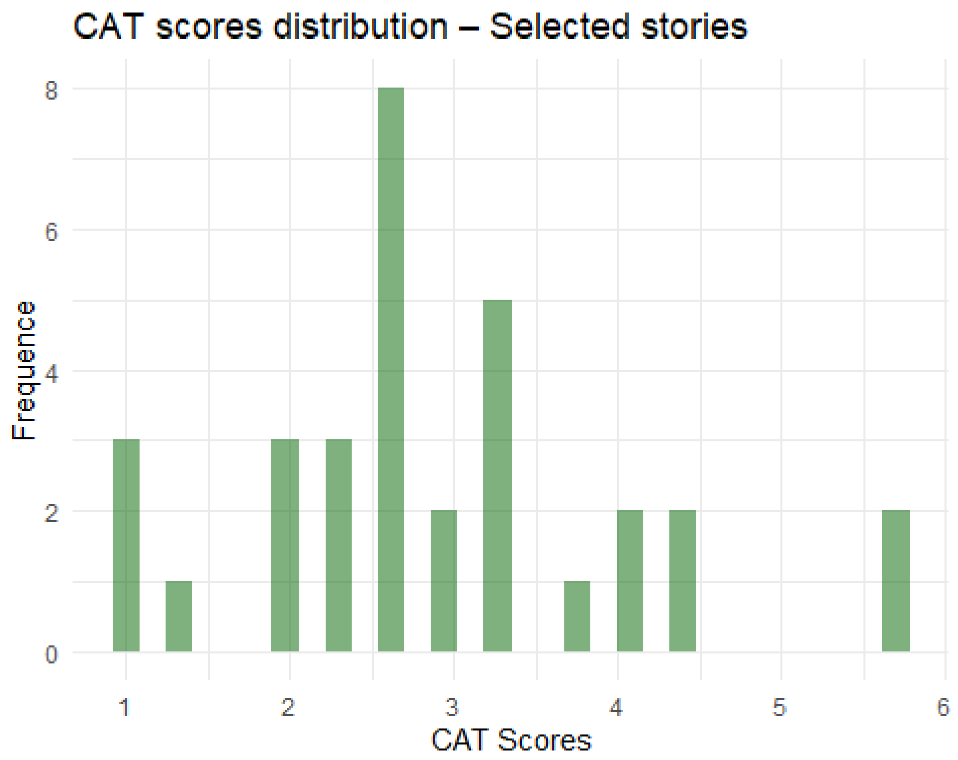

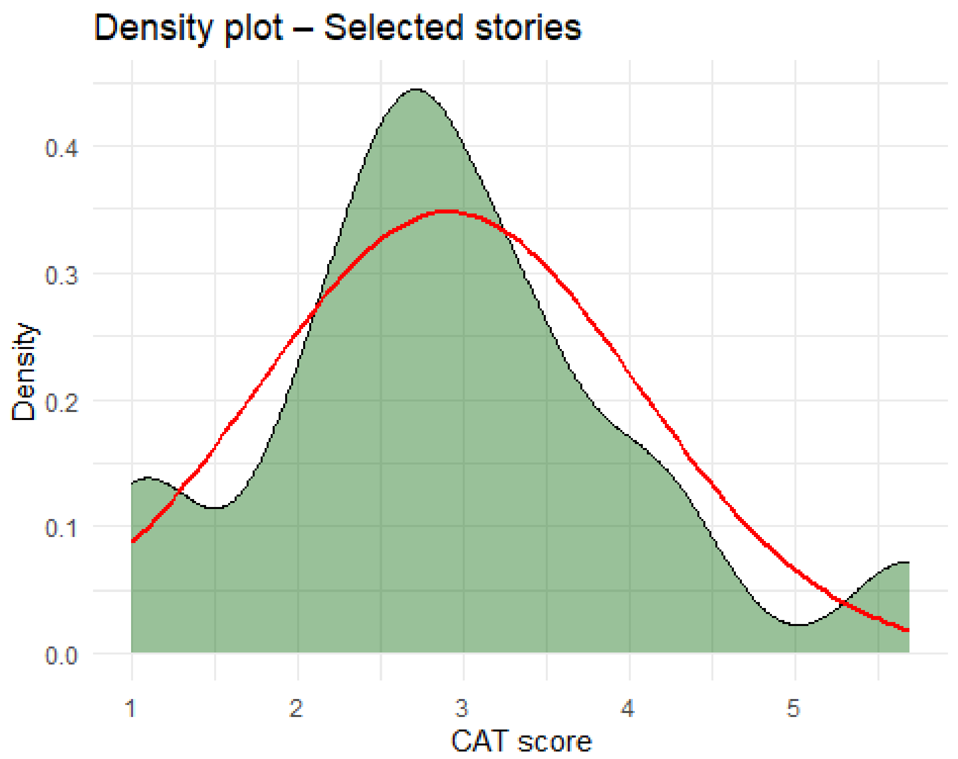

b. Selected stories grouped by set

Table 7: Descriptive statistics – Selected stories by set

set	N	meanCAT	sd	min	max	median
1A	8.00	2.75	1.00	1.00	4.33	2.83
1B	8.00	3.21	1.37	1.00	5.67	3.00
2A	8.00	2.59	0.78	1.30	4.00	2.70
2B	8.00	3.09	1.43	1.00	5.70	3.00

Table 8: Normality tests – Selected stories by set

set	KS	KS_p	SW	SW_p
1A	0.156	0.819	0.982	0.972
1B	0.222	0.293	0.937	0.586
2A	0.193	0.513	0.951	0.726
2B	0.137	0.928	0.980	0.964

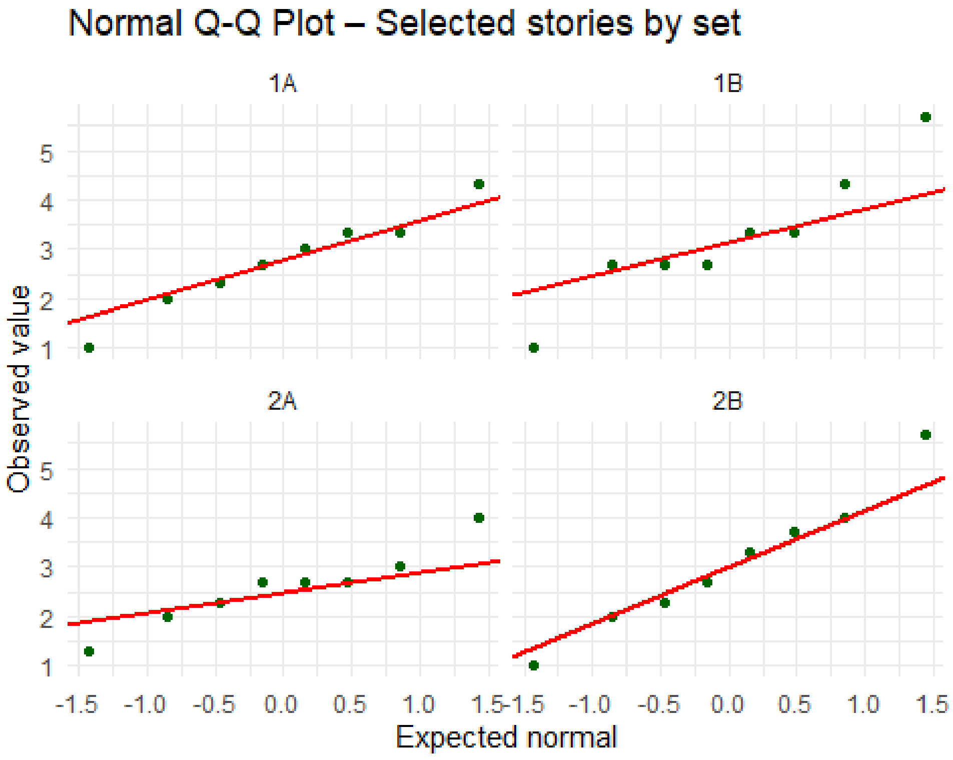

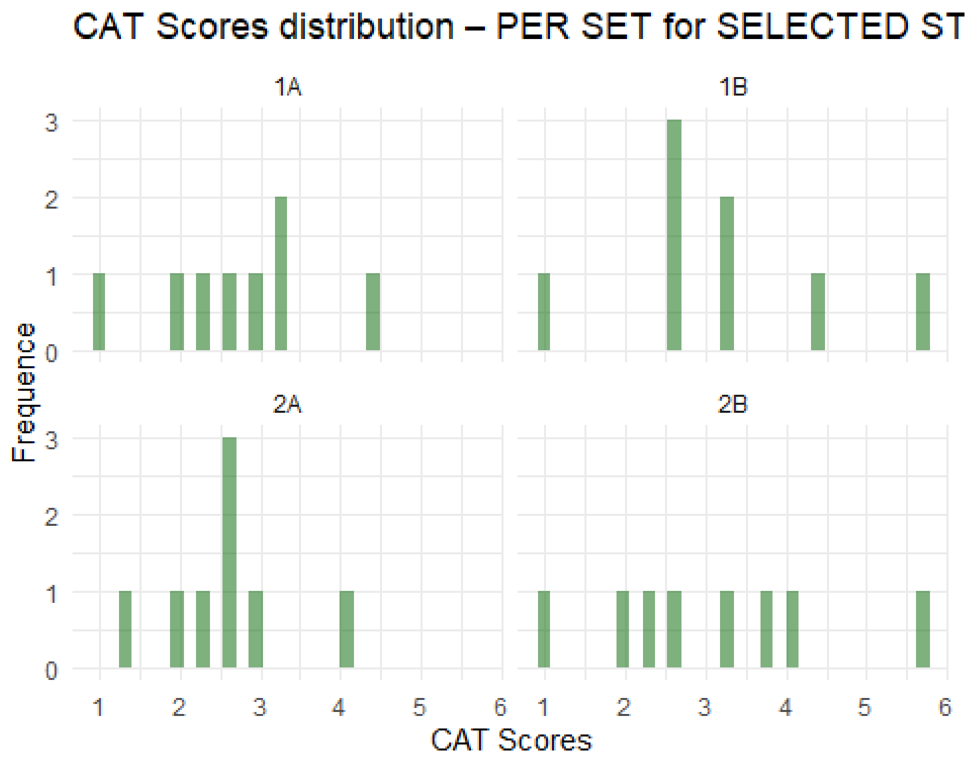

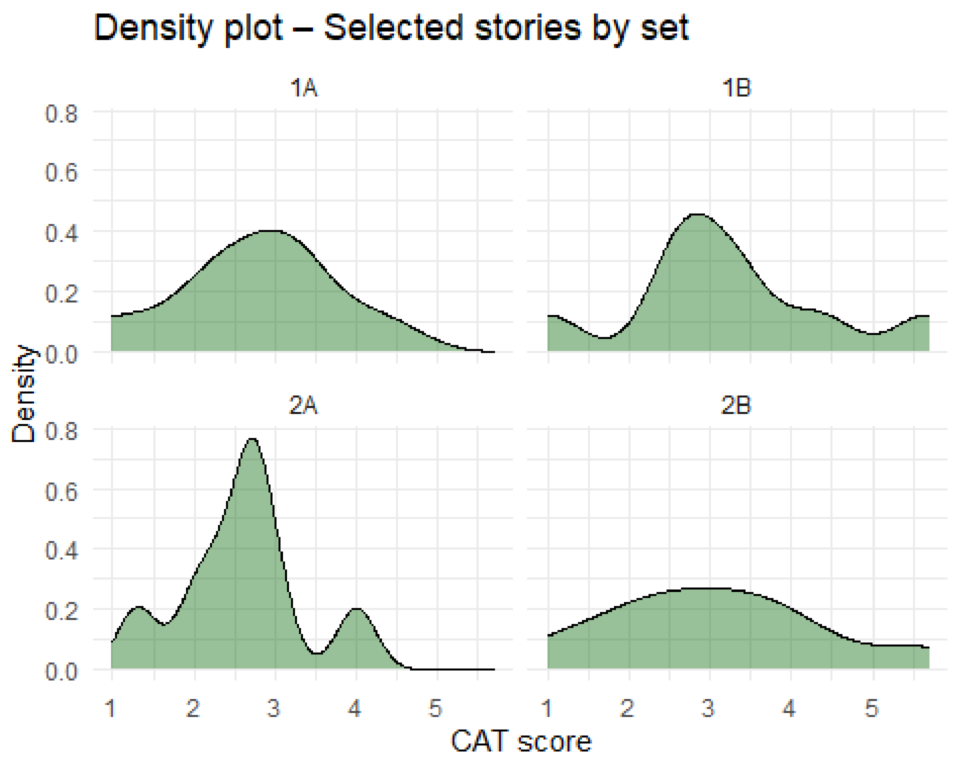

Table 9: Comparison of the density graphs of the CAT scores for each set of the selected stories (2) compared to the training set (0)

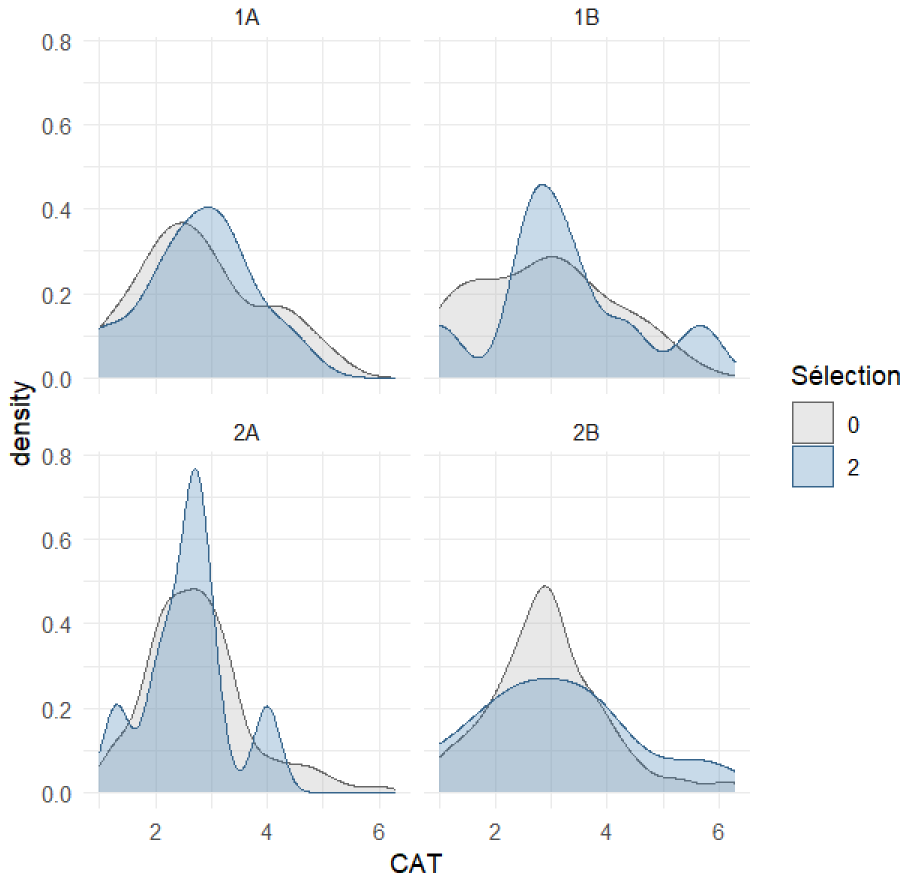

4. Prediction of CAT using RF

a. R Code to process RF models

```
library(googlesheets4)
library(dplyr)
library(randomForest)
library(openxlsx)
library(gt)
library(nortest)
# Connection to Google Sheet (url of the sheet containing the prepared data)
url <- "https://docs.google.com/spreadsheets/***URL***"
# Verification of the document and reading of the columns
df1 <- read_sheet(url)
str(df1)
# Listing of the columns following the name Pred_xxa (with x representing dou
ble numbers based on the number of stories used for training, and a being a c
ode letter to differentiate the sets between them)
pred_cols <- grep("^Pred_", names(df1), value = TRUE)
for (col in pred_cols) {
```

```
  message("Traitement de ", col, " ...")
  # Training set : lines or col == 1 and non-missing CAT
  train_data <- df1 %>%
    filter(.data[[col]] == 1, !is.na(CAT)) %>%
    select(DSI_mean, Length, CAT)
  if (nrow(train_data) < 10) {
    warning("not enough data to train the model on ", col)
    next
  }
  # Training Random Forest
  rf_model <- randomForest(CAT ~ DSI_mean + Length, data = train_data)
  # Lines to predict = col == NA
  rows_to_predict <- which(is.na(df1[[col]]))
  if (length(rows_to_predict) > 0) {
    new_data <- df1[rows_to_predict, c("DSI_mean", "Length")]
    preds <- predict(rf_model, newdata = new_data)
    # Rewrite the predictions in column Pred_xxx
    df1[rows_to_predict, col] <- preds
  }
}
# creation of output sheet
sheet_write(df1, ss = NULL, sheet = "Results")
```

b. Figure 1

```
library(tidyverse)
library(readxl)
Correlation_table <- read_excel("URL.xlsx")
df3 <- Correlation_table
# Split RF and Constant
df_RF <- df3 %>%
  filter(str_detect(Models, "^RF_")) %>%
  arrange(n_stories)
df_constantes <- df3 %>%
  filter(Models %in% c("MAoSS"))
# Constant values
MAoSS_value   <- df_constantes %>% filter(Models == "MAoSS") %>% pull(MCATr)
# Graph
breaks_x <- c(0, seq(0, max(df_RF$n_stories), by = 25))
ggplot() +
  # RF
  geom_line(data = df_RF,
            aes(x = n_stories, y = MCATr, color = "RF mean correlation"),
            linewidth = 1.1) +

  geom_point(data = df_RF,
             aes(x = n_stories, y = MCATr, color = "RF mean correlation"),
             size = 2) +
  # CI 95%
  geom_errorbar(data = df_RF,
```

```
                aes(x = n_stories,
                    ymin = `95%_MCATr_Lower`,
                    ymax = `95%_MCATr_Upper`,
                    color = "RF 95% CI"),
                width     =     5,
                linewidth = 0.8) +
  # Constant
  geom_hline(aes(yintercept = MAoSS_value, color = "MAoSS"), linetype = "dott
ed", linewidth = 1) +
  geom_hline(aes(yintercept = 0.70, color = "Threshold 0.70"), linewidth = 1)
+
  # Axes
  scale_x_continuous(
    breaks = breaks_x,
    limits = c(0, max(df_RF$n_stories))
  ) +
  scale_y_continuous(
    breaks = seq(0.5, 1, by = 0.1),
    minor_breaks = seq(0, 1, by = 0.05)
  ) +
  # Legend
  scale_color_manual(
    name = "Legend",
    values = c(
      "RF mean correlation" = "#1f4e79",
      "RF 95% CI" = "#1f4e79",
      "MAoSS" = "brown",
      "Threshold 0.70" = "red"
    )
  ) +
  labs(
    title = "",
    x = "Training set size",
    y = "Mean correlation with CAT scores"
  ) +
  theme_minimal(base_size = 14) +
  theme(
    plot.title = element_text(face = "bold", size = 16),
    panel.grid.minor = element_line(color = "grey90"),
    legend.position = "right"
  )
```

c. Calculating MAE s RMSE

```
library(dplyr)
library(stringr)
library(purrr)
library(tidyr)
```

```r
library(ggplot2)
library(haven)
library(DT)
MDPI_RFpred_results <- read_sav("data.sav")
df2 <- MDPI_RFpred_results
# Standardisation CAT + Pred_XXa + MAoSS
pred_cols <- grep("^Pred_", names(df2), value = TRUE)
df2_std <- df2 %>%
  mutate(
    CAT_std = as.numeric(scale(CAT)),
    across(all_of(pred_cols), ~ as.numeric(scale(.x)), .names = "std_{.col}")
,
    std_MAoSS = as.numeric(scale(MAoSS))
  )
compute_errors <- function(y_true, y_pred) {
  mae  <- mean(abs(y_true - y_pred), na.rm = TRUE)
  rmse <- sqrt(mean((y_true - y_pred)^2, na.rm = TRUE))
  return(c(MAE = mae, RMSE = rmse))
}
# Results for every Pred_XXa
results_pred <- map_dfr(
  pred_cols,
  ~ {
    pred_vec <- df2_std[[paste0("std_", .x)]]
    vals <- compute_errors(df2_std$CAT_std, pred_vec)
    tibble(
      Model = .x,
      MAE = vals["MAE"],
      RMSE = vals["RMSE"]
    )
  }
)
## Results for MAoSS
maoss_vals <- compute_errors(df2_std$CAT_std, df2_std$std_MAoSS)

results_maoss <- tibble(
  Model = "MAoSS",
  MAE = maoss_vals["MAE"],
  RMSE = maoss_vals["RMSE"]
)
## Fusion
results_std <- bind_rows(results_pred, results_maoss)
results_grouped <- results_std %>%
  filter(Model != "MAoSS") %>%
  mutate(
    Step = str_extract(Model, "(?<=Pred_)[0-9]+") |> as.numeric()
  ) %>%
  group_by(Step) %>%
  summarise(
    MAE = mean(MAE),
```

```
    RMSE = mean(RMSE)
  )
maoss_row <- results_std %>%
  filter(Model == "MAoSS") %>%
  mutate(Step = "MAoSS") %>%
  select(Step, MAE, RMSE)
results_grouped2 <- bind_rows(
  results_grouped %>% mutate(Step = as.character(Step)),
  maoss_row
)
step_levels <- c(sort(as.numeric(results_grouped$Step)), "MAoSS")
results_grouped_long <- results_grouped2 %>%
  pivot_longer(cols = c(MAE, RMSE), names_to = "Metric", values_to = "Value")
#Plot creation
ggplot(results_grouped_long,
       aes(x = factor(Step, levels = step_levels),
           y = Value, fill = Metric)) +
  geom_col(position = "dodge") +
  theme_minimal() +
  labs(
    title = "Mean error per training set + MAoSS (standardized)",
    x = "Step",
    y = "Error"
  )
#Tables print
results_std %>%
  gt() %>%
  tab_header(title = "Errors per model (standardized)") %>%
  fmt_number(columns = c(MAE, RMSE), decimals = 3)
results_grouped2 %>%
  gt() %>%
```

```
  tab_header(title = "Mean errors per step + MAoSS") %>%
  fmt_number(columns = c(MAE, RMSE), decimals = 3)
```

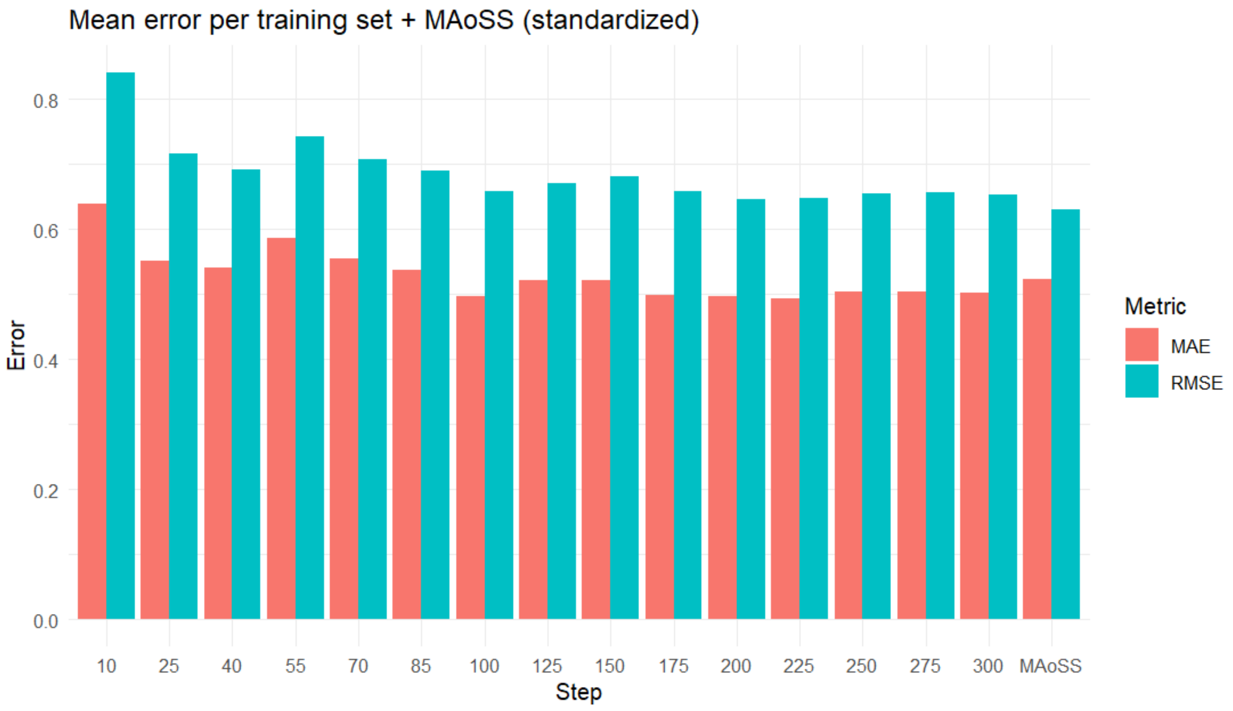

Table 11: Errors per model (standardized)

Model	MAE	RMSE
Pred_10a	0.665	0.867
Pred_10b	0.680	0.852
Pred_10c	0.731	0.963
Pred_10d	0.578	0.777
Pred_10e	0.557	0.789
Pred_10f	0.662	0.822
Pred_10g	0.556	0.773
Pred_10h	0.585	0.787
Pred_10i	0.668	0.839
Pred_10j	0.715	0.943
Pred_25a	0.583	0.777
Pred_25b	0.549	0.683
Pred_25c	0.513	0.652
Pred_25d	0.492	0.681
Pred_25e	0.613	0.758
Pred_25f	0.509	0.709
Pred_25g	0.584	0.765
Pred_25h	0.681	0.841
Pred_25i	0.484	0.619
Pred_25j	0.501	0.675
Pred_40a	0.597	0.774
Pred_40b	0.593	0.736
Pred_40c	0.568	0.700
Pred_40d	0.537	0.733
Pred_40e	0.587	0.686
Pred_40f	0.439	0.581
Pred_40g	0.520	0.682
Pred_40h	0.569	0.713
Pred_40i	0.456	0.576
Pred_40j	0.532	0.736
Pred_55a	0.603	0.762
Pred_55b	0.674	0.815
Pred_55c	0.690	0.818
Pred_55d	0.728	0.873
Pred_55e	0.549	0.722

Model	MAE	RMSE
Pred_55f	0.542	0.702
Pred_55g	0.548	0.705
Pred_55h	0.422	0.632
Pred_55i	0.535	0.691
Pred_55j	0.570	0.705
Pred_70a	0.553	0.707
Pred_70b	0.572	0.765
Pred_70c	0.571	0.714
Pred_70d	0.534	0.649
Pred_70e	0.526	0.691
Pred_70f	0.505	0.630
Pred_70g	0.701	0.833
Pred_70h	0.546	0.679
Pred_70i	0.536	0.713
Pred_70j	0.505	0.693
Pred_85a	0.603	0.742
Pred_85b	0.515	0.650
Pred_85c	0.536	0.691
Pred_85d	0.611	0.746
Pred_85e	0.619	0.759
Pred_85f	0.562	0.744
Pred_85g	0.510	0.638
Pred_85h	0.494	0.693
Pred_85i	0.460	0.633
Pred_85j	0.464	0.602
Pred_100a	0.459	0.620
Pred_100b	0.506	0.682
Pred_100c	0.459	0.590
Pred_100d	0.514	0.680
Pred_100e	0.512	0.666
Pred_100f	0.509	0.663
Pred_100g	0.513	0.667
Pred_100h	0.517	0.727
Pred_100i	0.523	0.680
Pred_100j	0.447	0.603

Model	MAE	RMSE
Pred_125a	0.575	0.737
Pred_125b	0.498	0.661
Pred_125c	0.493	0.609
Pred_125d	0.508	0.681
Pred_125e	0.528	0.685
Pred_125f	0.512	0.690
Pred_125g	0.476	0.602
Pred_125h	0.533	0.681
Pred_125i	0.523	0.655
Pred_125j	0.566	0.707
Pred_150a	0.543	0.674
Pred_150b	0.596	0.718
Pred_150c	0.473	0.622
Pred_150d	0.409	0.577
Pred_150e	0.555	0.702
Pred_150f	0.581	0.744
Pred_150g	0.546	0.758
Pred_150h	0.460	0.618
Pred_150i	0.540	0.701
Pred_150j	0.508	0.687
Pred_175a	0.515	0.720
Pred_175b	0.519	0.700
Pred_175c	0.464	0.615
Pred_175d	0.475	0.667
Pred_175e	0.520	0.695
Pred_175f	0.443	0.590
Pred_175g	0.543	0.693
Pred_175h	0.500	0.632
Pred_175i	0.503	0.631
Pred_175j	0.496	0.644
Pred_200a	0.458	0.645
Pred_200b	0.489	0.647
Pred_200c	0.467	0.601
Pred_200d	0.497	0.632
Pred_200e	0.514	0.667

Model	MAE	RMSE
Pred_200f	0.599	0.721
Pred_200g	0.424	0.570
Pred_200h	0.570	0.733
Pred_200i	0.495	0.638
Pred_200j	0.458	0.606
Pred_225a	0.498	0.658
Pred_225b	0.492	0.652
Pred_225c	0.523	0.688
Pred_225d	0.406	0.542
Pred_225e	0.423	0.560
Pred_225f	0.523	0.694
Pred_225g	0.509	0.658
Pred_225h	0.550	0.729
Pred_225i	0.506	0.688
Pred_225j	0.499	0.615
Pred_250a	0.521	0.675
Pred_250b	0.531	0.689
Pred_250c	0.518	0.640
Pred_250d	0.485	0.655
Pred_250e	0.447	0.617
Pred_250f	0.501	0.647
Pred_250g	0.524	0.673
Pred_250h	0.543	0.680
Pred_250i	0.460	0.636
Pred_250j	0.503	0.637
Pred_275a	0.494	0.635
Pred_275b	0.503	0.686
Pred_275c	0.556	0.738
Pred_275d	0.500	0.634
Pred_275e	0.522	0.671
Pred_275f	0.482	0.639
Pred_275g	0.462	0.601
Pred_275h	0.482	0.637
Pred_275i	0.549	0.703
Pred_275j	0.488	0.619

Model	MAE	RMSE
Pred_300a	0.527	0.671
Pred_300b	0.523	0.682
Pred_300c	0.508	0.671
Pred_300d	0.481	0.616
Pred_300e	0.504	0.659
Pred_300f	0.494	0.632
Pred_300g	0.489	0.643
Pred_300h	0.506	0.661
Pred_300i	0.491	0.647
Pred_300j	0.498	0.642
MAoSS	0.522	0.630

Table 12: Mean errors per step + MAoSS

Step	MAE	RMSE
10	0.640	0.841
25	0.551	0.716
40	0.540	0.692
55	0.586	0.743
70	0.555	0.707
85	0.537	0.690
100	0.496	0.658
125	0.521	0.671
150	0.521	0.680
175	0.498	0.659
200	0.497	0.646
225	0.493	0.648
250	0.503	0.655
275	0.504	0.656
300	0.502	0.652
MAoSS	0.522	0.630

5. Code and outputs for the RF simulations including the Storyboard item as input variables

a. Calculating the correlation with CAT

```
library(tidyverse)
library(purrr)
library(readxl)
library(gt)

MDPI_RFpred_results5 <- read_xlsx("MDPI_data/MDPI_RFpred+set_results.xlsx")
df5_pred <- MDPI_RFpred_results5
# Identify prediction columns
pred_cols5 <- df5_pred %>%
  select(starts_with("Pred_")) %>%
  colnames()
# Compute correlations for each Pred_XXa
correlations5 <- map_df(pred_cols5, function(col) {
  x <- df5_pred[[col]]
  y <- df5_pred$CAT
  complete_idx <- complete.cases(x, y)
  if (sum(complete_idx) < 2) {
    tibble(variable = col, correlation = NA_real_)
  } else {
    tibble(variable = col, correlation = cor(x[complete_idx], y[complete_idx]
))
  }
})
# Extract numeric group (XX)
correlations5 <- correlations5 %>%
  mutate(
    group = as.numeric(str_extract(variable, "(?<=Pred_)\\d+"))
  )
# Summary table
cor_summary5 <- correlations5 %>%
  group_by(group) %>%
  summarise(
    mean_correlation = mean(correlation, na.rm = TRUE),
    n_models = sum(!is.na(correlation))
  ) %>%
  arrange(group)
# Display table
cor_summary5 %>%
  gt() %>%
  tab_header(title = "Mean correlation with CAT per group – df5 version") %>%
  fmt_number(columns = c(mean_correlation), decimals = 3) %>%
  tab_style(
    style = cell_fill(color = "#f7f7f7"),
```

```
    locations = cells_body()
  )
```

Table 13: Mean correlation with CAT per group

group	mean_correlation	n_models
10	0.582	10
25	0.721	10
40	0.734	10
55	0.701	10
70	0.738	10
85	0.745	10
100	0.788	10
125	0.775	10
150	0.761	10
175	0.787	10
200	0.792	10
225	0.799	10
250	0.786	10
275	0.797	10
300	0.793	10

b. Calculating MAE s RMSE

```
library(dplyr)
library(stringr)
library(purrr)
library(tidyr)
library(ggplot2)
library(readxl)
library(DT)
library(gt)
MDPI_RFpred_results <- read_xlsx("MDPI_data/MDPI_RFpred+set_results.xlsx")
df5 <- MDPI_RFpred_results
# Standardisation CAT + Pred_XXa + MAoSS
pred_cols <- grep("^Pred_", names(df5), value = TRUE)
df5_std <- df5 %>%
  mutate(
    CAT_std = as.numeric(scale(CAT)),
    across(all_of(pred_cols), ~ as.numeric(scale(.x)), .names = "std_{.col}")
,
    std_MAoSS = as.numeric(scale(MAoSS))
  )
compute_errors <- function(y_true, y_pred) {
  mae  <- mean(abs(y_true - y_pred), na.rm = TRUE)
  rmse <- sqrt(mean((y_true - y_pred)^2, na.rm = TRUE))
```

```r
  return(c(MAE = mae, RMSE = rmse))
}
# Results for every Pred_XXa
results_pred5 <- map_dfr(
  pred_cols,
  ~ {
    pred_vec <- df5_std[[paste0("std_", .x)]]
    vals <- compute_errors(df5_std$CAT_std, pred_vec)
    tibble(
      Model = .x,
      MAE = vals["MAE"],
      RMSE = vals["RMSE"]
    )
  }
)
# Results for MAoSS
maoss_vals5 <- compute_errors(df5_std$CAT_std, df5_std$std_MAoSS)

results_maoss5 <- tibble(
  Model = "MAoSS",
  MAE = maoss_vals5["MAE"],
  RMSE = maoss_vals5["RMSE"]
)
# Fusion
results_std5 <- bind_rows(results_pred5, results_maoss5)
results_grouped5 <- results_std5 %>%
  filter(Model != "MAoSS") %>%
  mutate(
    Step = str_extract(Model, "(?<=Pred_)[0-9]+") |> as.numeric()
  ) %>%
  group_by(Step) %>%
  summarise(
    MAE = mean(MAE),
    RMSE = mean(RMSE)
  )
maoss_row5 <- results_std5 %>%
  filter(Model == "MAoSS") %>%
  mutate(Step = "MAoSS") %>%
  select(Step, MAE, RMSE)
results_grouped25 <- bind_rows(
  results_grouped5 %>% mutate(Step = as.character(Step)),
  maoss_row5
)
step_levels5 <- c(sort(as.numeric(results_grouped5$Step)), "MAoSS")
results_grouped_long5 <- results_grouped25 %>%
  pivot_longer(cols = c(MAE, RMSE), names_to = "Metric", values_to = "Value")
# Plot creation
ggplot(results_grouped_long5,
       aes(x = factor(Step, levels = step_levels5),
           y = Value, fill = Metric)) +
```

```
  geom_col(position = "dodge") +
  theme_minimal() +
  labs(
    title = "Mean error per training set + MAoSS (standardized) – df5",
    x = "Step",
    y = "Error"
  )
# Tables print
results_std5 %>%
  gt() %>%
  tab_header(title = "Errors per model (standardized) – df5") %>%
  fmt_number(columns = c(MAE, RMSE), decimals = 3)
results_grouped25 %>%
  gt() %>%
```

```
  tab_header(title = "Mean errors per step + MAoSS - df5") %>%
  fmt_number(columns = c(MAE, RMSE), decimals = 3)
```

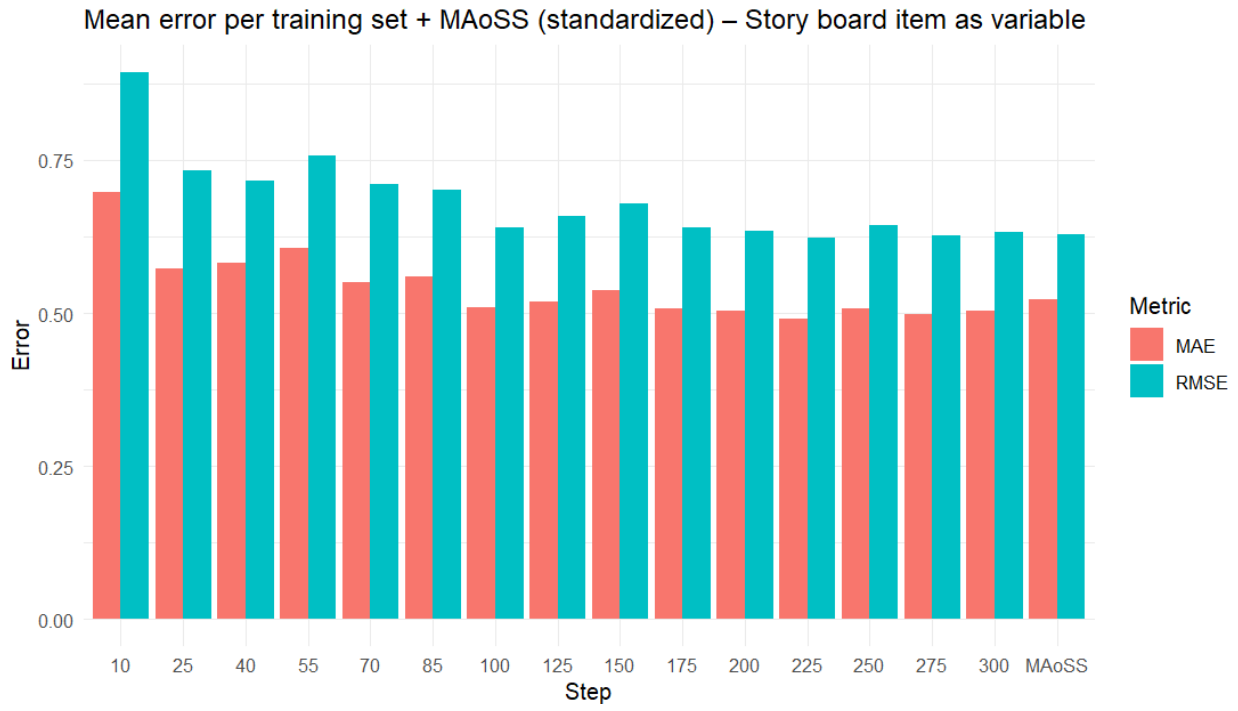

Table 14: Errors per model (standardized)

Model	MAE	RMSE
Pred_10a	0.785	0.947
Pred_10b	0.739	0.960
Pred_10c	0.756	1.060
Pred_10d	0.618	0.839
Pred_10e	0.575	0.781
Pred_10f	0.631	0.816
Pred_10g	0.623	0.747
Pred_10h	0.707	0.844
Pred_10i	0.697	0.909
Pred_10j	0.839	1.039
Pred_25a	0.607	0.765
Pred_25b	0.519	0.679
Pred_25c	0.627	0.823
Pred_25d	0.457	0.628
Pred_25e	0.632	0.774
Pred_25f	0.535	0.695
Pred_25g	0.575	0.737
Pred_25h	0.609	0.755
Pred_25i	0.599	0.742
Pred_25j	0.577	0.739
Pred_40a	0.547	0.742
Pred_40b	0.595	0.741
Pred_40c	0.592	0.732
Pred_40d	0.534	0.706
Pred_40e	0.605	0.715
Pred_40f	0.552	0.617
Pred_40g	0.595	0.717
Pred_40h	0.633	0.766
Pred_40i	0.501	0.593
Pred_40j	0.676	0.824
Pred_55a	0.651	0.774
Pred_55b	0.704	0.895
Pred_55c	0.583	0.729
Pred_55d	0.671	0.856
Pred_55e	0.545	0.702
Pred_55f	0.555	0.662
Pred_55g	0.651	0.782
Pred_55h	0.543	0.720

Model	MAE	RMSE
Pred_55i	0.516	0.663
Pred_55j	0.641	0.790
Pred_70a	0.535	0.695
Pred_70b	0.511	0.683
Pred_70c	0.577	0.721
Pred_70d	0.617	0.728
Pred_70e	0.484	0.664
Pred_70f	0.503	0.670
Pred_70g	0.555	0.728
Pred_70h	0.521	0.678
Pred_70i	0.647	0.811
Pred_70j	0.555	0.735
Pred_85a	0.565	0.719
Pred_85b	0.506	0.650
Pred_85c	0.577	0.725
Pred_85d	0.605	0.780
Pred_85e	0.619	0.762
Pred_85f	0.604	0.719
Pred_85g	0.495	0.646
Pred_85h	0.539	0.674
Pred_85i	0.555	0.708
Pred_85j	0.523	0.638
Pred_100a	0.480	0.606
Pred_100b	0.547	0.711
Pred_100c	0.513	0.634
Pred_100d	0.466	0.586
Pred_100e	0.456	0.575
Pred_100f	0.507	0.623
Pred_100g	0.570	0.694
Pred_100h	0.587	0.734
Pred_100i	0.487	0.619
Pred_100j	0.490	0.613
Pred_125a	0.550	0.700
Pred_125b	0.629	0.741
Pred_125c	0.557	0.693
Pred_125d	0.433	0.609
Pred_125e	0.510	0.668
Pred_125f	0.533	0.710

Model	MAE	RMSE
Pred_125g	0.484	0.600
Pred_125h	0.457	0.553
Pred_125i	0.469	0.591
Pred_125j	0.570	0.711
Pred_150a	0.512	0.592
Pred_150b	0.549	0.698
Pred_150c	0.520	0.666
Pred_150d	0.501	0.665
Pred_150e	0.572	0.724
Pred_150f	0.537	0.677
Pred_150g	0.557	0.697
Pred_150h	0.558	0.697
Pred_150i	0.563	0.715
Pred_150j	0.508	0.663
Pred_175a	0.554	0.689
Pred_175b	0.516	0.679
Pred_175c	0.470	0.600
Pred_175d	0.509	0.663
Pred_175e	0.481	0.619
Pred_175f	0.442	0.559
Pred_175g	0.505	0.599
Pred_175h	0.581	0.693
Pred_175i	0.530	0.679
Pred_175j	0.487	0.629
Pred_200a	0.518	0.639
Pred_200b	0.565	0.709
Pred_200c	0.528	0.639
Pred_200d	0.484	0.593
Pred_200e	0.507	0.642
Pred_200f	0.567	0.663
Pred_200g	0.446	0.570
Pred_200h	0.500	0.685
Pred_200i	0.505	0.637
Pred_200j	0.426	0.560
Pred_225a	0.546	0.675
Pred_225b	0.466	0.618
Pred_225c	0.528	0.640
Pred_225d	0.451	0.578

Model	MAE	RMSE
Pred_225e	0.441	0.561
Pred_225f	0.506	0.640
Pred_225g	0.525	0.649
Pred_225h	0.449	0.639
Pred_225i	0.500	0.613
Pred_225j	0.492	0.617
Pred_250a	0.524	0.696
Pred_250b	0.542	0.651
Pred_250c	0.478	0.604
Pred_250d	0.519	0.660
Pred_250e	0.464	0.583
Pred_250f	0.505	0.641
Pred_250g	0.488	0.609
Pred_250h	0.541	0.687
Pred_250i	0.483	0.616
Pred_250j	0.540	0.680
Pred_275a	0.467	0.588
Pred_275b	0.454	0.619
Pred_275c	0.478	0.627
Pred_275d	0.563	0.688
Pred_275e	0.536	0.655
Pred_275f	0.518	0.617
Pred_275g	0.480	0.611
Pred_275h	0.481	0.636
Pred_275i	0.483	0.592
Pred_275j	0.519	0.639
Pred_300a	0.528	0.651
Pred_300b	0.576	0.710
Pred_300c	0.519	0.640
Pred_300d	0.494	0.598
Pred_300e	0.501	0.637
Pred_300f	0.474	0.621
Pred_300g	0.455	0.584
Pred_300h	0.482	0.613
Pred_300i	0.512	0.636
Pred_300j	0.498	0.629
MAoSS	0.522	0.630

Table 15: Mean errors per step + MAoSS

Step	MAE	RMSE
10	0.697	0.894
25	0.574	0.734
40	0.583	0.715
55	0.606	0.757
70	0.551	0.711
85	0.559	0.702
100	0.510	0.640
125	0.519	0.658
150	0.538	0.679
175	0.507	0.641
200	0.504	0.634
225	0.490	0.623
250	0.508	0.643
275	0.498	0.627
300	0.504	0.632
MAoSS	0.522	0.630

6. Code and outputs for the RF simulations with residualized CAT scores (for length) as criterion

a. Calculating the correlation with CAT_rez

```
# Pred.
library(googlesheets4)
library(dplyr)
library(randomForest)
library(openxlsx)
library(gt)
library(nortest)
# Connection to Google Sheet (url of the sheet containing the prepared data)
url <- ""
# Verification of the document and reading of the columns
df1 <- read_sheet(url)
str(df1)
# Listing of the columns following the name Pred_xxa (with x representing dou
ble numbers based on the number of stories used for training, and a being a c
ode letter to differentiate the sets between them)
pred_cols <- grep("^Pred_", names(df1), value = TRUE)
for     (col     in     pred_cols)     {
  message("Traitement de ", col, " ...")
```

```
  # Training set : lines or col == 1 and non-missing CAT
  train_data <- df1 %>%
    filter(.data[[col]] == 1, !is.na(CAT)) %>%
    select(DSI_mean, Length, CAT, CAT_rez)
  if (nrow(train_data) < 10) {
    warning("not enough data to train the model on ", col)
    next
  }
  # Training Random Forest
  rf_model <- randomForest(CAT_rez ~ DSI_mean + Length, data = train_data)
  # Lines to predict = col == NA
  rows_to_predict <- which(is.na(df1[[col]]))
  if (length(rows_to_predict) > 0) {
    new_data <- df1[rows_to_predict, c("DSI_mean", "Length")]
    preds <- predict(rf_model, newdata = new_data)
    # Rewrite the predictions in column Pred_xxx
    df1[rows_to_predict, col] <- preds
  }
}
# creation of a new google sheet
sheet_write(df1, ss = NULL, sheet = "Results")


# Corr.
library(tidyverse)
library(readxl)
library(gt)
library(stringr)
library(tidyr)
library(ggplot2)
library(writexl)
# Load data
df <- read_excel("MDPI_data/RF_output_CATrez.xlsx")
# Identify prediction columns
pred_cols <- df %>%
  select(starts_with("Pred_")) %>%
  colnames()
# Safe correlation function
safe_cor <- function(x, y) {
  idx <- complete.cases(x, y)
  if (sum(idx) < 2) return(NA_real_)
  cor(x[idx], y[idx])
}
# Compute correlations for Pred_ variables
correlations <- tibble(
  variable = pred_cols,
  correlation = map_dbl(pred_cols, ~ safe_cor(df[[.x]], df$CAT_rez)),
  group = as.numeric(str_extract(pred_cols, "(?<=Pred_)\\d+"))
)
# Summary by group (Pred_ only)
```

```r
cor_summary_models <- correlations %>%
  group_by(group) %>%
  summarise(
    mean_correlation = mean(correlation, na.rm = TRUE),
    n_models = sum(!is.na(correlation)),
    .groups = "drop"
  )
# Add MAoSS correlation
maoss_cor <- tibble(
  group = "MAoSS",
  mean_correlation = safe_cor(df$MAoSS, df$CAT_rez),
  n_models = 1
)
cor_summary_all <- bind_rows(
  cor_summary_models %>% mutate(group = as.character(group)),
  maoss_cor
)
# Display correlation summary table
cor_summary_all %>%
  gt() %>%
  tab_header(title = "Mean Correlation with CAT_rez per Group + MAoSS") %>%
  fmt_number(columns = mean_correlation, decimals = 3) %>%
```

```
  tab_style(
    style = cell_fill(color = "#f7f7f7"),
    locations = cells_body()
  )
```

Table 26: Mean Correlation with CAT_rez per Group + MAoSS

group	mean_correlation	n_models
10	0.241	10
25	0.270	10
40	0.375	10
55	0.238	10
70	0.238	10
85	0.266	10
100	0.304	10
125	0.193	10
150	0.247	10
175	0.293	10
200	0.247	10
225	0.273	10
250	0.227	10
275	0.200	10
300	0.213	10
MAoSS	0.511	1

b. Calculating MAE s RMSE

```
# Standardize variables for error metrics
df_std <- df %>%
  mutate(
    CAT_std = as.numeric(scale(CAT_rez)),
    across(all_of(pred_cols), ~ as.numeric(scale(.x)), .names = "std_{.col}")
,
    std_MAoSS = as.numeric(scale(MAoSS))
  )
# Error metrics function
compute_errors <- function(y_true, y_pred) {
  mae  <- mean(abs(y_true - y_pred), na.rm = TRUE)
  rmse <- sqrt(mean((y_true - y_pred)^2, na.rm = TRUE))
  c(MAE = mae, RMSE = rmse)
}
# Errors for each Pred_ model
results_pred <- map_dfr(
```

```
  pred_cols,
  ~ {
    pred_vec <- df_std[[paste0("std_", .x)]]
    vals <- compute_errors(df_std$CAT_std, pred_vec)
    tibble(
      Model = .x,
      MAE = vals["MAE"],
      RMSE = vals["RMSE"]
    )
  }
)
# Errors for MAoSS
maoss_vals <- compute_errors(df_std$CAT_std, df_std$std_MAoSS)
results_maoss <- tibble(
  Model = "MAoSS",
  MAE = maoss_vals["MAE"],
  RMSE = maoss_vals["RMSE"]
)
# Combine all error results
results_std <- bind_rows(results_pred, results_maoss)
# Grouped errors by step (Pred_ only)
results_grouped <- results_std %>%
  filter(Model != "MAoSS") %>%
  mutate(
    Step = str_extract(Model, "(?<=Pred_)[0-9]+") |> as.numeric()
  ) %>%
  group_by(Step) %>%
  summarise(
    MAE = mean(MAE),
    RMSE = mean(RMSE),
    .groups = "drop"
  )
# Add MAoSS row to grouped errors
maoss_row <- results_std %>%
  filter(Model == "MAoSS") %>%
  mutate(Step = "MAoSS") %>%
  select(Step, MAE, RMSE)
results_grouped2 <- bind_rows(
  results_grouped %>% mutate(Step = as.character(Step)),
  maoss_row
)
# Prepare long format for plotting errors
step_levels <- c(sort(as.numeric(results_grouped$Step)), "MAoSS")
results_grouped_long <- results_grouped2 %>%
  pivot_longer(cols = c(MAE, RMSE), names_to = "Metric", values_to = "Value")
# Plot mean correlation per group + MAoSS
p1 <- ggplot(cor_summary_all,
             aes(x = factor(group, levels = c(sort(unique(cor_summary_all$gro
up[cor_summary_all$group != "MAoSS"])), "MAoSS")),
                 y = mean_correlation)) +
```

```
  geom_col(fill = "#4C72B0") +
  theme_minimal() +
  labs(title = "Mean Correlation per Group + MAoSS",
       x = "Group",
       y = "Correlation")
# Plot mean error per step + MAoSS
ggplot(results_grouped_long,
             aes(x = factor(Step, levels = step_levels),
                 y = Value,
                 fill = Metric)) +
  geom_col(position = "dodge") +
  theme_minimal() +
  labs(title = "Mean Error per Step + MAoSS (Standardized) – CAT-rez as outpu
t",
       x = "Step",
       y = "Error")
# Display error tables
results_std %>%
  gt() %>%
  tab_header(title = "Errors per Model (Standardized)") %>%
  fmt_number(columns = c(MAE, RMSE), decimals = 3)

results_grouped2 %>%
  gt() %>%
```

```
  tab_header(title = "Mean Errors per Step + MAoSS") %>%
  fmt_number(columns = c(MAE, RMSE), decimals = 3)
```

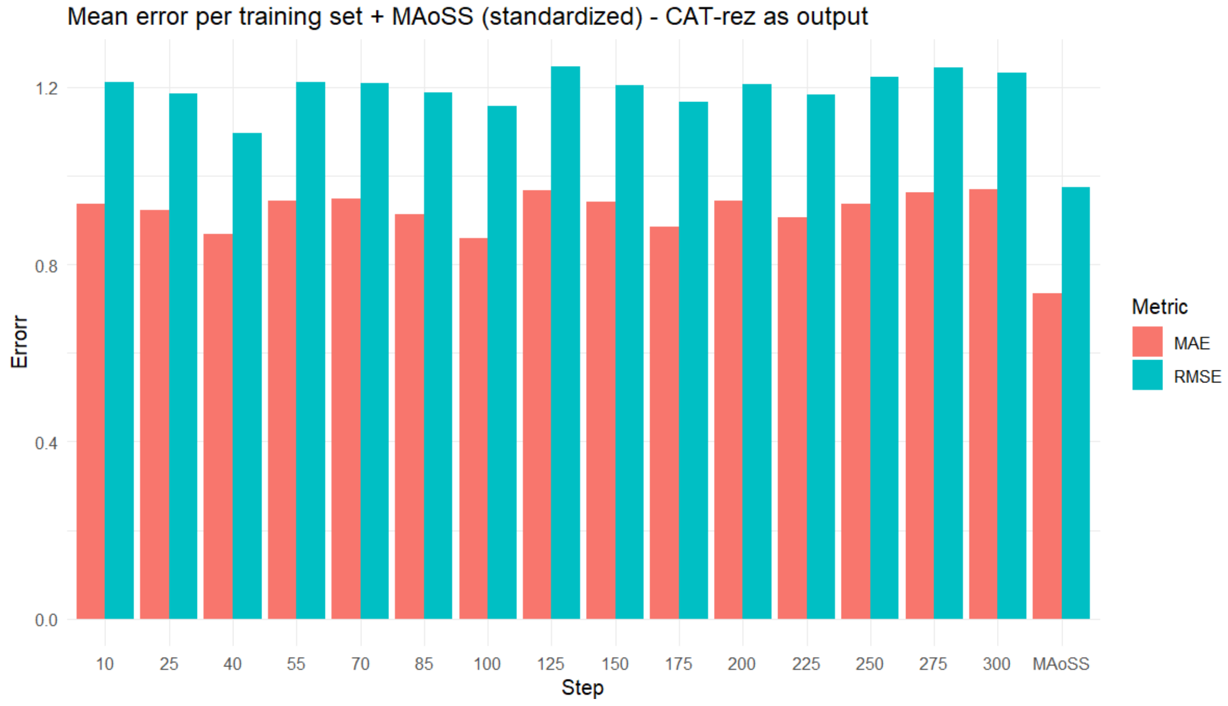

Table 37: Errors per Model (Standardized)

Model	MAE	RMSE
Pred_10a	0.818	1.099
Pred_10b	0.964	1.233
Pred_10c	0.931	1.226
Pred_10d	0.947	1.269
Pred_10e	0.869	1.192
Pred_10f	0.901	1.134
Pred_10g	0.886	1.190
Pred_10i	0.998	1.201
Pred_10j	1.019	1.207
Pred_10k	1.039	1.357
Pred_25a	1.050	1.328
Pred_25b	0.790	1.023
Pred_25c	0.983	1.262
Pred_25d	1.019	1.307
Pred_25e	0.848	1.144
Pred_25f	0.892	1.171
Pred_25g	0.874	1.121
Pred_25i	1.041	1.307
Pred_25j	0.789	0.985
Pred_25k	0.938	1.194
Pred_40a	0.962	1.272
Pred_40b	0.826	1.055
Pred_40c	1.067	1.208
Pred_40d	0.906	1.186
Pred_40e	0.863	1.049
Pred_40f	0.784	0.999
Pred_40g	0.793	1.052
Pred_40i	0.848	1.026
Pred_40j	0.772	0.981
Pred_40k	0.859	1.135
Pred_55a	0.950	1.181
Pred_55b	1.057	1.260
Pred_55c	0.979	1.190
Pred_55d	0.986	1.375
Pred_55e	1.120	1.444

Model	MAE	RMSE
Pred_55f	0.949	1.230
Pred_55g	0.876	1.142
Pred_55i	0.737	1.095
Pred_55j	0.894	1.093
Pred_55k	0.882	1.089
Pred_70a	0.888	1.151
Pred_70b	0.896	1.181
Pred_70c	1.018	1.211
Pred_70d	0.874	1.094
Pred_70e	0.882	1.110
Pred_70f	0.853	1.096
Pred_70g	1.272	1.530
Pred_70i	0.920	1.121
Pred_70j	0.937	1.339
Pred_70k	0.936	1.246
Pred_85a	1.024	1.248
Pred_85b	0.893	1.173
Pred_85c	0.971	1.234
Pred_85d	0.971	1.217
Pred_85e	1.055	1.336
Pred_85f	0.952	1.265
Pred_85g	0.736	0.936
Pred_85i	0.949	1.323
Pred_85j	0.768	1.051
Pred_85k	0.812	1.084
Pred_100a	0.650	0.988
Pred_100b	0.792	1.063
Pred_100c	0.881	1.117
Pred_100d	0.923	1.242
Pred_100e	0.796	1.060
Pred_100f	0.894	1.195
Pred_100g	0.912	1.246
Pred_100i	0.973	1.271
Pred_100j	0.883	1.216
Pred_100k	0.894	1.179

Model	MAE	RMSE
Pred_125a	0.997	1.285
Pred_125b	0.950	1.231
Pred_125c	0.886	1.166
Pred_125d	1.139	1.431
Pred_125e	1.039	1.330
Pred_125f	0.938	1.226
Pred_125g	0.842	1.093
Pred_125i	0.860	1.086
Pred_125j	0.987	1.325
Pred_125k	1.036	1.286
Pred_150a	1.059	1.297
Pred_150b	1.040	1.212
Pred_150c	0.848	1.145
Pred_150d	0.723	1.012
Pred_150e	0.971	1.192
Pred_150f	1.075	1.319
Pred_150g	0.992	1.302
Pred_150i	0.831	1.144
Pred_150j	0.955	1.274
Pred_150k	0.913	1.151
Pred_175a	0.941	1.350
Pred_175b	0.898	1.158
Pred_175c	0.894	1.145
Pred_175d	0.799	1.079
Pred_175e	1.005	1.285
Pred_175f	0.799	1.162
Pred_175g	0.898	1.148
Pred_175i	0.888	1.145
Pred_175j	0.980	1.247
Pred_175k	0.739	0.940
Pred_200a	0.801	1.100
Pred_200b	0.968	1.226
Pred_200c	0.875	1.130
Pred_200d	1.012	1.248
Pred_200e	1.011	1.248

Model	MAE	RMSE
Pred_200f	1.041	1.308
Pred_200g	0.859	1.080
Pred_200i	1.046	1.341
Pred_200j	0.856	1.140
Pred_200k	0.960	1.231
Pred_225a	0.854	1.061
Pred_225b	0.935	1.216
Pred_225c	0.853	1.135
Pred_225d	0.797	1.040
Pred_225e	0.788	1.077
Pred_225f	0.994	1.304
Pred_225g	0.981	1.309
Pred_225i	0.940	1.288
Pred_225j	1.019	1.291
Pred_225k	0.895	1.104
Pred_250a	1.009	1.294
Pred_250b	0.885	1.183
Pred_250c	0.993	1.209
Pred_250d	0.923	1.240
Pred_250e	0.856	1.180
Pred_250f	0.935	1.230
Pred_250g	0.960	1.248
Pred_250i	0.977	1.223
Pred_250j	0.856	1.208
Pred_250k	0.960	1.219
Pred_275a	0.894	1.190
Pred_275b	0.980	1.313
Pred_275c	1.054	1.336
Pred_275d	0.930	1.183
Pred_275e	1.046	1.322
Pred_275f	0.955	1.216
Pred_275g	0.913	1.163
Pred_275i	0.911	1.193
Pred_275j	1.020	1.334
Pred_275k	0.913	1.179

Model	MAE	RMSE
Pred_300a	1.026	1.299
Pred_300b	0.988	1.274
Pred_300c	0.987	1.302
Pred_300d	0.856	1.061
Pred_300e	1.004	1.260
Pred_300f	0.889	1.163
Pred_300g	1.002	1.222
Pred_300i	0.970	1.250
Pred_300j	0.951	1.236
Pred_300k	1.028	1.264
MAoSS	0.734	0.973

Table 48: Mean Errors per Step + MAoSS

Step	MAE	RMSE
10	0.937	1.211
25	0.922	1.184
40	0.868	1.096
55	0.943	1.210
70	0.947	1.208
85	0.913	1.187
100	0.860	1.158
125	0.967	1.246
150	0.941	1.205
175	0.884	1.166
200	0.943	1.205
225	0.906	1.182
250	0.935	1.223
275	0.962	1.243
300	0.970	1.233
MAoSS	0.734	0.973
